# Scattering interference signature of a pair density wave state in the cuprate pseudogap phase

**DOI:** 10.1038/s41467-021-26028-x

**Published:** 2021-10-19

**Authors:** Shuqiu Wang, Peayush Choubey, Yi Xue Chong, Weijiong Chen, Wangping Ren, H. Eisaki, S. Uchida, Peter J. Hirschfeld, J. C. Séamus Davis

**Affiliations:** 1 Clarendon Laboratory, University of Oxford, Oxford, UK; 2 Institut für Theoretische Physik III, Ruhr-Universität Bochum, Bochum, Germany; 3 Department of Physics, Indian Institute of Technology (Indian School of Mines), Dhanbad, Jharkhand India; 4 LASSP, Department of Physics, Cornell University, Ithaca, NY USA; 5 Institute of Advanced Industrial Science and Tech., Tsukuba, Ibaraki, Japan; 6 Department of Physics, University of Florida, Gainesville, FL USA; 7 Department of Physics, University College Cork, Cork, Ireland; 8 Max-Planck Institute for Chemical Physics of Solids, Dresden, Germany

**Keywords:** Electronic properties and materials, Quantum fluids and solids, Superconducting properties and materials

## Abstract

An unidentified quantum fluid designated the pseudogap (PG) phase is produced by electron-density depletion in the CuO_2_ antiferromagnetic insulator. Current theories suggest that the PG phase may be a pair density wave (PDW) state characterized by a spatially modulating density of electron pairs. Such a state should exhibit a periodically modulating energy gap ${\Delta }_{{{{{{\rm{P}}}}}}}({{{{{\boldsymbol{r}}}}}})$ in real-space, and a characteristic quasiparticle scattering interference (QPI) signature ${\Lambda }_{{{{{{\rm{P}}}}}}}({{{{{\boldsymbol{q}}}}}})$ in wavevector space. By studying strongly underdoped Bi_2_Sr_2_CaDyCu_2_O_8_ at hole-density ~0.08 in the superconductive phase, we detect the 8*a*_0_-periodic ${\Delta }_{{{{{{\rm{P}}}}}}}({{{{{\boldsymbol{r}}}}}})$ modulations signifying a PDW coexisting with superconductivity. Then, by visualizing the temperature dependence of this electronic structure from the superconducting into the pseudogap phase, we find the evolution of the scattering interference signature $\Lambda ({{{{{\boldsymbol{q}}}}}})$ that is predicted specifically for the temperature dependence of an 8*a*_0_-periodic PDW. These observations are consistent with theory for the transition from a PDW state coexisting with *d*-wave superconductivity to a pure PDW state in the Bi_2_Sr_2_CaDyCu_2_O_8_ pseudogap phase.

## Abstract (web summary)

The pseudogap phase in cuprate superconductors is predicted to be a pair density wave state (PDW) but experimental evidence has been lacking. Here, the authors detect the temperature evolution of energy gap modulations and scattering interference signature suggesting the Bi_2_Sr_2_CaDyCu_2_O_8_ pseudogap phase contains a PDW.

## Introduction

**Fig. 1:**
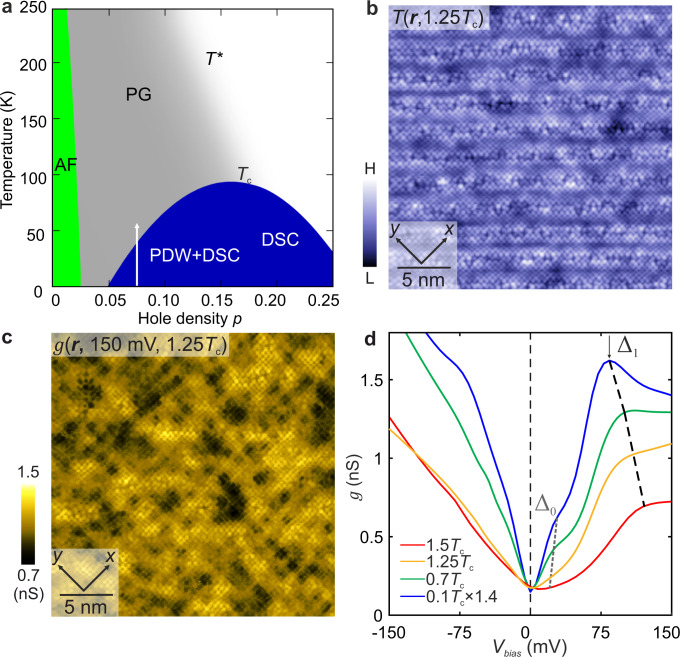
Temperature dependence of cuprate broken-symmetry states. **a** Schematic phase diagram of hole-doped cuprates. The Mott insulator phase with long range antiferromagnetic order (AF) is replaced by the pseudogap phase (PG) with increasing hole doping *p* below the onset temperature T*. The PG phase is characterized by the suppression of magnetic susceptibility, electronic specific heat, the c-axis conductivity and the average density of electronic states, and the appearance of a truncated Fermi surface. The d-symmetry Cooper-paired high-temperature superconductivity state (DSC) is indicated schematically in a blue “dome”. The range of temperature *T*, in which the PG state is studied in this paper is indicated by the white arrow. **b** Topograph *T*(***r***) at the BiO termination layer at *T* = 1.25*T*_c_ in the PG phase of Bi_2_Sr_2_CaDyCu_2_O_8_ for *p* ≈ 0.08. **c** Differential conductance map $g\left({{{{{\boldsymbol{r}}}}}},+150{{{{{\rm{mV}}}}}}\right)$ was obtained at the same field of view as (**b**) at *T* = 1.25*T*_c_ = 45 K. The *g*(***r***, E) manifests $\lambda =4{a}_{0}$ charge modulations. **d** Evolution of the spatially averaged tunneling conductance spectra of Bi_2_Sr_2_CaDyCu_2_O_8_ with increasing *T*, here characterized by *T*_c_. The gap ${\triangle }_{1}(T)$ is the energy of the coherence peak that is identified by a local maximum in *g*(V) for V>0 (indicated by a black vertical arrow). The energies ${\triangle }_{0}(T)$ (gray dashed line) are identified as the extinction energy of Bogoliubov quasiparticles (see movie S1). The two characteristic energies ${\triangle }_{0}\left(T\right)$ and ${\triangle }_{1}(T)$ appear more subtle at higher temperatures due to thermal broadening. Note the tunneling spectra at 4.2 K (≈ 0.1*T*_c_) is multiplied by 1.4.

Carrier-doped CuO_2_ sustains both high temperature superconductivity and the pseudogap quantum fluid, often simultaneously. Although the former is reasonably well understood, a decades-long effort by physicists to identify the latter^1,2^ has yet to bear fruit. The essential phenomenology of the pseudogap, while complex, is internally consistent. When *p* holes per unit-cell are introduced to CuO_2_, the antiferromagnetic insulator (AF) state disappears and the pseudogap (PG) emerges in the region p < p* and T < T*(p) (Fig. 1a). For $T\;\lesssim \;{T}^{\ast }\left(p\right)$, an energy gap ${\Delta }^{\ast }\left(p\right)$ depletes the spectrum of electronic states, and thus the magnetic susceptibility^3^ $\chi (T)$, the electronic specific heat^4^ $C(T)$, the c-axis conductivity^5,6^ $\rho (\omega ,T)$, and the average density of electronic states^7^ $N\left(E\right).$ In ***k***-space, there are four ${{{{{\boldsymbol{k}}}}}}{{{{{\boldsymbol{(}}}}}}E=0{{{{{\boldsymbol{)}}}}}}$ Fermi arcs^8^ neighboring ${{{{{\boldsymbol{k}}}}}}\approx (\pm \pi /2a_{0},\pm \pi /2a_{0})$, beyond which the ‘pseudogap’ ${\Delta }^{\ast }\left({{{{{\boldsymbol{k}}}}}}\right)\,$ opens^3,9,10^ near ${{{{{\boldsymbol{k}}}}}}\approx (\pm \pi /a_{0},0);(0,\pm \pi /a_{0})$. At extreme magnetic fields, tiny electron-like pockets with ***k***-space area ${A}_{k}\approx 7 \%$ of the CuO_2_ Brillouin zone, are detected^11^ in the pseudogap state. Probes of electrical and thermal transport in the pseudogap phase evidence electron-pairs without phase rigidity^12–14^. Translational symmetry breaking is widely reported^15–17^ to occur within the pseudogap phase; it is associated with charge density modulations of wavevectors ${{{{{\boldsymbol{Q}}}}}}\approx 2\pi /a_{0}\left(\pm 1/4,0\right);(0,\pm 1/4)$. A 90°-rotational (C_4_) symmetry breaking at ${{{{{\boldsymbol{Q}}}}}}$ = **0** and sometimes time-reversal symmetry breaking are also reported depending on materials and technique^18–22^. All these phenomena disappear^10,23,24^ near a critical hole density $p={p}^{\ast }$ which depends on material. The long-term challenge has been to identify a specific state of electronic matter that should exhibit all these properties simultaneously. A viable candidate has emerged recently^25–38^, the pair density wave state^39^.

A spatially homogeneous *d*-wave superconductor has an electron-pair potential or order parameter ${\Delta }_{d}({{{{{\boldsymbol{r}}}}}})={\Delta }_{0}{e}^{i\phi }$ with macroscopic quantum phase $\phi$ and critical temperature *T*_c_. By contrast, a PDW state has an order parameter ${\Delta }_{{{{{{\rm{P}}}}}}}({{{{{\boldsymbol{r}}}}}})$ that modulates spatially at wavevectors ${{{{{{\boldsymbol{Q}}}}}}}_{{{{{{\rm{P}}}}}}}$

1 $${\Delta }_{{{{{{\rm{P}}}}}}}({{{{{\boldsymbol{r}}}}}})=\left[\Delta \left({{{{{\boldsymbol{r}}}}}}\right){e}^{i{{{{{{\boldsymbol{Q}}}}}}}_{{{{{{\rm{P}}}}}}}\cdot {{{{{\boldsymbol{r}}}}}}}+{\Delta }^{\ast }\left({{{{{\boldsymbol{r}}}}}}\right){e}^{-i{{{{{{\boldsymbol{Q}}}}}}}_{{{{{{\rm{P}}}}}}}\cdot {{{{{\boldsymbol{r}}}}}}}\right]{e}^{i\theta }$$

with a macroscopic quantum phase $\theta$. In theory, such a state exhibits a particle-hole symmetric energy gap ${\Delta }_{{{{{{\rm{P}}}}}}}({{{{{\boldsymbol{k}}}}}})$ near the Brillouin zone (${{{{{\rm{BZ}}}}}}$) edges, with the ${\Delta }_{{{{{{\rm{P}}}}}}}({{{{{\boldsymbol{k}}}}}})=0$ points connected by extended ${{{{{\boldsymbol{k}}}}}}\left(E=0\right)$ Fermi arcs^8–10^. Of necessity, such a partial gap suppresses $N(E)$, $C(T)$, $\chi (T)$, and $\rho (\omega ,T)$. Moreover, a pure PDW is defined by a pair potential modulation as in equation (1) and exhibits a primary electron-pair density modulation ${{{{{\rho }}}}}_{{{{{{\rm{P}}}}}}}({{{{{\boldsymbol{r}}}}}})={{{{{\rho }}}}}_{{{{{{\rm{P}}}}}}}^{0}\left[{e}^{i2{{{{{{\boldsymbol{Q}}}}}}}_{{{{{{\rm{P}}}}}}}\cdot {{{{{\boldsymbol{r}}}}}}}+{e}^{-i2{{{{{{\boldsymbol{Q}}}}}}}_{{{{{{\rm{P}}}}}}}\cdot {{{{{\boldsymbol{r}}}}}}}\right]$ along with a collateral charge density modulation ${{{{{\rho }}}}}_{{{{{{\rm{C}}}}}}}({{{{{\boldsymbol{r}}}}}})={{{{{\rho }}}}}_{{{{{{\rm{C}}}}}}}^{0}\left[{e}^{i{{{{{{\boldsymbol{Q}}}}}}}_{{{{{{\rm{C}}}}}}}\cdot {{{{{\boldsymbol{r}}}}}}}+{e}^{-i{{{{{{\boldsymbol{Q}}}}}}}_{{{{{{\rm{C}}}}}}}\cdot {{{{{\boldsymbol{r}}}}}}}\right]$ with wavevector ${{{{{{\boldsymbol{Q}}}}}}}_{{{{{{\rm{C}}}}}}}=2{{{{{{\boldsymbol{Q}}}}}}}_{{{{{{\rm{P}}}}}}}$ (ref. ^39^). If the PDW is unidirectional, it necessarily breaks the rotation symmetry of the material at ***Q***=**0**, and if biaxial it can break time reversal symmetry^40^. PDW order very naturally produces Fermi arcs^26,30,41,42^. Finally, quasiparticles of the PDW should exhibit scattering interference signatures^35^ which are uniquely characteristic of that state.

While charge density modulations ${{{{{\rho }}}}}_{{{{{{\rm{C}}}}}}}({{{{{\boldsymbol{r}}}}}})$ are widely reported in the pseudogap phase^15–17^ it is unknown if electron-pair density ${{{{{\rho }}}}}_{{{{{{\rm{P}}}}}}}({{{{{\boldsymbol{r}}}}}})$ or electron-pair potential ${\Delta }_{{{{{{\rm{P}}}}}}}({{{{{\boldsymbol{r}}}}}})$ modulations exist therein. Whether the QPI signature ${\Lambda }_{{{{{{\rm{P}}}}}}}({{{{{\boldsymbol{q}}}}}})$ of a PDW occurs in the pseudogap phase is also unknown. Indeed, exploration of the pseudogap phase in search of a PDW poses severe experimental challenges. The modulating electron-pair density ρ_*P*_(***r***) which is iconic of the PDW state has been visualized directly by scanned Josephson tunneling microscopy^36,43^ but such experiments must be carried out at sub-kelvin temperatures where both sample and STM tip are superconducting. Another approach used in the superconductive phase has been to visualize signatures of the PDW electron-pair potential modulations^35,37,38^ ${\Delta }_{{{{{{\rm{P}}}}}}}({{{{{\boldsymbol{r}}}}}})$. But none of these experiments provide evidence on whether the pseudogap state in zero magnetic field is a PDW, because they were all carried out deep in the superconducting phase at temperatures $T\lesssim 0.1{T}_{c}$. At low temperatures but in high magnetic fields, both scanning tunneling microscopy and quantum oscillation studies report evidence for a PDW state^37,44^, implying that the relict of suppressed superconductivity is a PDW. Therefore, our objective is to visualize the evolution with temperature of electronic structure, especially ${\Delta }_{{{{{{\rm{P}}}}}}}({{{{{\boldsymbol{r}}}}}})$ and ${\Lambda }_{{{{{{\rm{P}}}}}}}({{{{{\boldsymbol{q}}}}}})$ from the superconducting into the zero-field pseudogap phase of strongly underdoped Bi_2_Sr_2_CaDyCu_2_O_8_.

## Results

### Modeling the temperature dependence of the PDW state

For theoretical guidance, we use a quantitative, atomic-scale model for PDW state based upon CuO_2_ electronic structure and the *t-J* Hamiltonian,

2 $$H=-\mathop{\sum}\limits_{\left(i,j\right),\sigma \,}{P}_{G}{t}_{{ij}}\left({c}_{i\sigma }^{{{\dagger}} }{c}_{j\sigma }+h.c.\right){P}_{G}+J\mathop{\sum}\limits_{ < i,j > }{{{{{{\bf{S}}}}}}}_{i}\cdot{{{{{{\bf{S}}}}}}}_{j}\,$$

Here, the electron hopping rates between nearest neighbor (NN) and next-nearest neighbor (NNN) Cu ${d}_{{x}^{2}-{y}^{2}}$ orbitals are *t* and *t’*, respectively, the onsite repulsive energy $U\to \infty$, thus the antiferromagnetic exchange interactions *J*, and the operator *P*_*G*_ eliminates all doubly-occupied orbitals. A renormalized mean-field theory (RMFT) approximation then replaces *P*_*G*_ with site-specific and bond-specific renormalization factors ${g}_{i,j}^{t}$ and ${g}_{i,j}^{s}$ based on the average number of charge and spin configurations permissible^34,35^. The resulting Hamiltonian is decoupled into a diagonalizable mean-field approximation using on-site hole density ${\delta }_{i}$, bond field ${{{{{{\rm{\chi }}}}}}}_{{ij}\sigma }$, and electron-pair potential ${\Delta }_{{ij}\sigma }\,$. This mean field *t-J* Hamiltonian has a uniform *d*-wave superconducting (DSC) state as its ground state, but PDW and DSC states are extremely close in energy, as has also been shown elsewhere^45–47^. Our approach is to find metastable configurations of PDW states and study their signatures in STM. To this end, the RMFT equations are initialized with the electron pair potential fields modulating at wavevector ${{{{{{\boldsymbol{Q}}}}}}}_{{{{{{\rm{P}}}}}}}=(\pm 1/8,0)2\pi /{a}_{0}$, as suggested by recent observations of electron-pair density modulating at 2***Q***_P_^36^ and energy-gap modulations at ${{{{{{\boldsymbol{Q}}}}}}}_{{{{{{\rm{P}}}}}}}$ at zero-magnetic field^38^ as well as in magnetic fields^37^. Moreover because there is little evidence of any long-range magnetic order coexisting with charge modulations in Bi_2_Sr_2_CaCu_2_O_8_ at any temperatures, we constrain the RMFT solutions to non-magnetic modulating states only, thus, excluding ($\pi ,\pi$) spin density wave order and stripe order. In the self-consistent solution wavefunction ${\Psi }_{0}({{{{{\boldsymbol{r}}}}}})$ of this broken-symmetry state then predicts the net charge on each Cu site ${\delta }_{i}=1- < {\Psi }_{0}\left|{\sum }_{\sigma }{n}_{i\sigma }\right|{\Psi }_{0} >$, the bond-field between adjacent sites *i,j* ${{{{{{\rm{\chi }}}}}}}_{{ij}\sigma }= < {\Psi }_{0}|{c}_{i\sigma }^{{{\dagger}} }{c}_{j\sigma }|{\Psi }_{0} >$, and the electron-pair field on the bond between adjacent sites *i,j* ${\Delta }_{{ij}\sigma }=\sigma\, < \, {\Psi }_{0}|{c}_{i\sigma }{c}_{j\bar{\sigma }}|{\Psi }_{0} >$. Finally, because experimental visualizations are carried out at the crystal termination BiO layer of Bi_2_Sr_2_CaCu_2_O_8_, Cu ${d}_{{x}^{2}-{y}^{2}}$ Wannier functions ${{{{{{\rm{W}}}}}}}_{i}\left({{{{{\boldsymbol{r}}}}}}\right)$ and lattice Green’s function ${{{{{{\rm{G}}}}}}}_{{ij}\sigma }({{{{{\rm{E}}}}}})$ are used to generate the ***r***-space Green’s functions ${{{{{{\rm{G}}}}}}}_{\sigma }\left({{{{{\boldsymbol{r}}}}}},{{{{{\rm{E}}}}}}\right)={\sum }_{{ij}}{{{{{{{\rm{G}}}}}}}_{{ij}\sigma }({{{{{\rm{E}}}}}}){{{{{\rm{W}}}}}}}_{i}\left({{{{{\boldsymbol{r}}}}}}\right){{{{{{\rm{W}}}}}}}_{j}^{\ast }\left({{{{{\boldsymbol{r}}}}}}\right)$ everywhere at a height 0.4 nm above BiO terminal plane^48^. Thus, the atomically resolved density of electronic states $N({{{{{\boldsymbol{r}}}}}},E) = {\sum }_{\sigma } -\frac{1}{\pi}{{{{{\rm{Im}}}}}}\; {{{{{\rm{G}}}}}}_{\sigma }({{{{{\boldsymbol{r}}}}}},{{{{{\rm{E}}}}}})$ at the BiO termination surface of Bi_2_Sr_2_CaCu_2_O_8_ is predicted for the case where the adjacent CuO_2_ crystal layer sustains a ${{{{{\rm{\lambda }}}}}}=8{a}_{0}$ PDW (Supplementary Note 1).

**Fig. 2:**
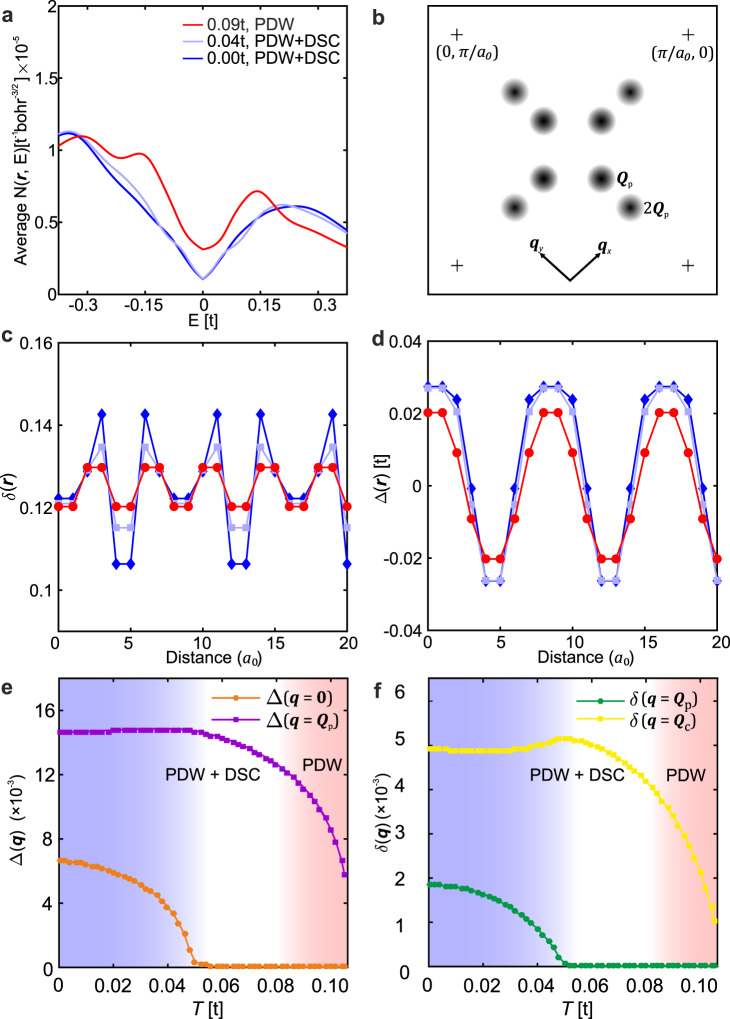
Predicted temperature-evolution of the average local density of states (*N*(*r, E*)), hole density (*δ*), and d-wave gap order parameter (Δ) for the PDW state. **a** Continuum LDOS $N({{{{{\boldsymbol{r}}}}}},{{{{{\rm{E}}}}}})$ spatially averaged over a period of PDW (8${a}_{0}$) at low-temperatures (*T*=0 and *T*=0.04*t*) and pure PDW state at a high temperature (*T*=0.09*t*) obtained using parameter set doping *p* = 0.125, *t* = 400 meV, *t’* = -0.3*t* and *J* = 0.3*t*. PDW+DSC state exhibits V-shaped nodal LDOS due to the presence of the DSC component. Pure PDW state has Bogoliubov-Fermi pockets (in contrast to nodes in PDW+DSC state), which leads to a large *E* = 0 LDOS. The LDOS is obtained after the effects of linear inelastic scattering $i{0}^{+}+i\varGamma$ is incorporated, where $\varGamma =\alpha {|E|}$ and *α* = 0.25 using the experimental fits in ref. ^55^. A non-zero LDOS at zero-bias in PDW+DSC state is a consequence of the finite artificial broadening $i{0}^{+}$. **b** q-space schematic showing the most prominent wavevectors ${{{{{{\boldsymbol{Q}}}}}}}_{{{{{{\rm{P}}}}}}}$ = [(±1/8, 0); (0, ±1/8)]2π/*a*_0_ and ${2{{{{{\boldsymbol{Q}}}}}}}_{{{{{{\rm{P}}}}}}}$ appearing in the Fourier transform of the mean-fields and other related quantities. **c** Spatial variation of hole density (δ) in PDW+DSC state (at *T*=0 and *T*=0.04*t*) and pure PDW state (at *T*=0.09*t*). Hole density modulates with a periodicity of 8${a}_{0}$ in PDW+DSC state due to presence of the DSC component and a periodicity of 4${a}_{0}$ in pure PDW state due to the absence of the DSC component, as expected from Ginzburg-Landau theories. **d** Spatial variation of d-wave gap order parameter in PDW+DSC state (at *T*=0 and *T*=0.04*t*) and pure PDW state (at *T* = 0.09*t*) exhibiting 8${a}_{0}$-periodic modulations corresponding to the PDW component of the gap. **e** Temperature evolution of the uniform (${{{{{\boldsymbol{q}}}}}}={{{\bf{0}}}}$) and PDW (${{{{{\boldsymbol{q}}}}}}={{{{{{\boldsymbol{Q}}}}}}}_{{{{{{\rm{P}}}}}}}$) components of the d-wave gap order parameter in PDW+DSC state (0<*T*<0.085*t*) and pure PDW state (0.085*t*<*T*<0.11*t*). The uniform component of the gap decreases sharply with temperature, becoming negligibly small but finite compared to the PDW component for 0.05*t*<*T*<0.085*t*. This ‘fragile PDW+DSC’ state is shown in white background. For *T*>0.085*t* PDW+DSC state becomes unstable and only pure PDW state (shown in pink background) exists as a stable solution of the RMFT equations. **f** Temperature evolution of the ${{{{{\boldsymbol{q}}}}}}={{{{{{\boldsymbol{Q}}}}}}}_{{{{{{\rm{P}}}}}}}$ and ${{{{{\boldsymbol{q}}}}}}={{{{{{\boldsymbol{Q}}}}}}}_{{{{{{\rm{C}}}}}}}=2{{{{{{\boldsymbol{Q}}}}}}}_{{{{{{\rm{P}}}}}}}$ components of hole density (δ) in PDW+DSC and pure PDW state in the same temperature range as in (**e**). The ${{{{{\boldsymbol{q}}}}}}={{{{{{\boldsymbol{Q}}}}}}}_{{{{{{\rm{P}}}}}}}$ component mirrors the temperature evolution of the uniform component of the gap in panel (**e**), as expected from Ginzburg-Landau theories. ${{{{{\boldsymbol{q}}}}}}={{{{{{\boldsymbol{Q}}}}}}}_{{{{{{\rm{C}}}}}}}$ component is the dominant component at all temperature leading to 4${a}_{0}$–periodic charge density wave.

From this theory, Fig. 2a shows the average $N\left({{{{{\boldsymbol{r}}}}}},E\right)$ at height ~4 Å above the BiO termination in Bi_2_Sr_2_CaCu_2_O_8_ for the PDW state coexisting with d-wave superconductivity (PDW+DSC state) at low-temperatures and pure PDW state at a higher temperature. The PDW+DSC state shows a V-shaped *N(E)* due to presence of nodes in DSC state. With increasing temperature, the uniform component of the pair potential decreases (Supplementary Note 1 and Fig. 2e) and gap scales corresponding to DSC (${\Delta }_{0}$) and PDW (${\Delta }_{1}$) components can be identified as a shoulder feature and a coherence peak, respectively (light-blue curve corresponding to *T* = 0.04*t*). Nodal points disappear in transition from PDW+DSC state to PDW state at higher temperatures leading to a large zero-energy *N(E)* in the latter (red curve corresponding to *T* = 0.09*t*) (Supplementary Note 1). Thus, a finite zero energy density-of-states is a natural property of a PDW state. These features agree with the experimental findings (Fig. 1d). However, the spectral gap defined by the position of the *E*>0 coherence peak reduces in the high-temperature PDW state due to the reduced ${\Delta }_{{ij}\sigma }$. We believe this discrepancy is a result of an inadequate treatment of self-energy effects in the current renormalized mean field theory, including the assumption of temperature independent Gutzwiller factors (Supplementary Note 1). Figure 2b shows the most prominent Fourier components of the mean-fields in PDW+DSC and PDW states namely ${{{{{{\boldsymbol{Q}}}}}}}_{{{{{{\rm{P}}}}}}}{{{{{\boldsymbol{=}}}}}}\left(\pm 2\pi /8{a}_{0},0\right)$ and ${{{{{{\boldsymbol{Q}}}}}}}_{{{{{{\rm{C}}}}}}}=2{{{{{{\boldsymbol{Q}}}}}}}_{{{{{{\rm{P}}}}}}}$. All mean-fields, including the hole density and the *d*-wave gap order parameter^34^ shown in Figs. 2c and 2d, respectively, exhibit periodicity of $8{a}_{0}$ in the PDW+DSC state at low temperatures. However, due to the absence of the uniform component (${{{{{\boldsymbol{q}}}}}}={{{\bf{0}}}}$) in pure PDW state, density-like quantities are $4{a}_{0}$ periodic as predicted by Ginzburg-Landau theories^39^ (Fig. 2c). We note that the bond field χ_ij_ is a density-like quantity too and exhibits a behavior very similar to the hole density (Supplementary Note 1). The temperature dependence of the uniform and PDW (${{{{{\boldsymbol{q}}}}}}={{{{{{\boldsymbol{Q}}}}}}}_{{{{{{\rm{P}}}}}}}$) components are shown in Fig. 2e. With increasing temperature, the uniform component of the gap, which corresponds to DSC in the PDW+DSC state, decreases rapidly and becomes negligibly small compared to the PDW component in the temperature range 0.05*t*<*T*<0.085*t*, but does not vanish. We have verified that a converged nonzero solution for Δ(${{{{{\boldsymbol{q}}}}}}={{{{{\bf{0}}}}}}$), ‘fragile PDW+DSC’ state’, exists in this region (white background in Fig. 2e-f). For *T*>0.085*t*, the PDW+DSC solution of the RMFT equations becomes unstable and the pure PDW state is the only stable solution for a modulated state (pink background in Fig. 2e–f) (Supplementary Note 1). The temperature dependence of ${{{{{\boldsymbol{q}}}}}}={{{{{{\boldsymbol{Q}}}}}}}_{{{{{{\rm{P}}}}}}}$ and ${{{{{\boldsymbol{q}}}}}}={{{{{{\boldsymbol{Q}}}}}}}_{{{{{{\rm{C}}}}}}}$ components of the hole density is shown in Fig. 2f. We find that the ${{{{{\boldsymbol{q}}}}}}={{{{{{\boldsymbol{Q}}}}}}}_{{{{{{\rm{P}}}}}}}$ component of the charge density is dominant at all temperatures and the ${{{{{\boldsymbol{q}}}}}}={{{{{{\boldsymbol{Q}}}}}}}_{{{{{{\rm{P}}}}}}}$ component exhibits essentially the same temperature dependence as the uniform component of the gap order parameter. This is in agreement with Ginzburg–Landau theory^39^ and experimental observation^37,38,43^ that a PDW generated charge density wave (CDW) state will have ${{{{{\boldsymbol{q}}}}}}={{{{{{\boldsymbol{Q}}}}}}}_{{{{{{\rm{P}}}}}}}\,$and ${{{{{\boldsymbol{q}}}}}}={{{{{{\boldsymbol{Q}}}}}}}_{{{{{{\rm{C}}}}}}}=2{{{{{{\boldsymbol{Q}}}}}}}_{{{{{{\rm{P}}}}}}}$ components that are related to the uniform ($\varDelta ({{{\bf{0}}}})$) and PDW ($\Delta ({{{{{{\boldsymbol{Q}}}}}}}_{{{{{{\rm{P}}}}}}})$) components of the gap order parameter as $\delta ({{{{{{\boldsymbol{Q}}}}}}}_{{{{{{\rm{P}}}}}}})\propto (\Delta ({{{{{{\bf{0}}}}}}}){\Delta (-{{{{{{\boldsymbol{Q}}}}}}}_{{{{{{\rm{P}}}}}}})}^{\ast }+\Delta ({{{{{{\boldsymbol{Q}}}}}}}_{{{{{{\rm{P}}}}}}}){\Delta ({{{{{\bf{0}}}}}})}^{\ast })$ and $\delta ({{{{{{\boldsymbol{Q}}}}}}}_{{{{{{\rm{C}}}}}}})\propto \Delta ({{{{{{\boldsymbol{Q}}}}}}}_{{{{{{\rm{P}}}}}}}){\Delta (-{{{{{{\boldsymbol{Q}}}}}}}_{{{{{{\rm{P}}}}}}})}^{\ast }.$ The self-consistent PDW solutions are found to exist in a hole doping range 0.06 < *p* < 0.14 for all temperatures considered.

We explore these predictions using strongly underdoped Bi_2_Sr_2_CaDyCu_2_O_8_ samples with resistive transition temperature ${T}_{{{{{{\rm{c}}}}}}}= 37\pm 3\,{{{{{\rm{K}}}}}}$ and $p\cong 0.08$ as shown schematically by the white arrow in Fig. 1a. These samples are cleaved in cryogenic vacuum at $T\approx 4.2\,{{{{{\rm{K}}}}}}$ and inserted to the instrument. Measurements are carried out at a sequence of temperatures from ${0.1T}_{{{{{{\rm{c}}}}}}}\le T\le 1.5{T}_{{{{{{\rm{c}}}}}}}$ spanning the range from the superconducting to well into the pseudogap phase. The topographic images $T({{{{{\boldsymbol{r}}}}}})$ of the FOV studied versus temperature are taken using the experimental methods described in “Methods” section and presented in Supplementary Fig. 4. Both the tip-sample differential tunneling current $I\left({{{{{\boldsymbol{r}}}}}},V\right)$ and conductance ${dI}/{dV}({{{{{\boldsymbol{r}}}}}},E={eV})\equiv g({{{{{\boldsymbol{r}}}}}},V)$ are measured at bias voltage *V=E/e* and with sub-angstrom spatial resolution. Because the density of electronic states $N({{{{{\boldsymbol{r}}}}}},E)$ is related to the differential conductance as $g({{{{{\boldsymbol{r}}}}}},E)\,{\propto }N({{{{{\boldsymbol{r}}}}}},E)[{I}_{s}/{\int }_{0}^{{{eV}}_{s}}N({{{{{\boldsymbol{r}}}}}},{E}^{{\prime} })d{E}^{{\prime} }]$, where *I*_*s*_ and *V*_*s*_ are arbitrary set-point parameters and the denominator ${\int }_{0}^{{{eV}}_{s}}N({{{{{\boldsymbol{r}}}}}},E^{\prime} ){dE}^{\prime}$ is unknown, valid imaging of $N({{{{{\boldsymbol{r}}}}}},E)$ is intractable. However, one can suppress these serious systematic “set-point” errors by using $R\left({{{{{\boldsymbol{r}}}}}},E\right)=I({{{{{\boldsymbol{r}}}}}},E)/I\left({{{{{\boldsymbol{r}}}}}},-E\right)$ or $Z\left({{{{{\boldsymbol{r}}}}}},E\right)=g({{{{{\boldsymbol{r}}}}}},E)/g\left({{{{{\boldsymbol{r}}}}}},-E\right)$ so that distances, modulation wavelengths and spatial-phases can be measured accurately. Furthermore, Bogoliubov quasiparticle scattering interference (BQPI) occurs when an impurity atom scatters quasiparticles, which interfere to produce characteristic modulations of $\delta N({{{{{\boldsymbol{r}}}}}},{E})$ surrounding each scattering site. The Fourier transform of $\delta N({{{{{\boldsymbol{r}}}}}},{E})$, $\delta N\left({{{{{\boldsymbol{q}}}}}},{E}\right),$ then exhibits intensity maxima at a set of wavevectors ${{{{{{\boldsymbol{q}}}}}}}_{i}\,$connecting regions of high joint-density-of-states. Local maxima in $Z\left({{{{{\boldsymbol{q}}}}}},E\right)$ therefore reveal the sets of energy dispersive wavevectors ${{{{{{\boldsymbol{q}}}}}}}_{i}(E)$ generated by the scattering interference. An efficient synopsis of these complex phenomena can then be achieved^10^ by using $\Lambda \left({{{{{\boldsymbol{q}}}}}},\Delta \right)={\sum }_{E\cong 0}^{\Delta }Z({{{{{\boldsymbol{q}}}}}},E)$, which provides a characteristic “fingerprint” of whatever ordered state, e.g. CDW or PDW, controls the ${{{{{{\boldsymbol{q}}}}}}}_{i}(E)$.

### Evolution of energy gap modulations from superconductive to pseudogap phase

**Fig. 3:**
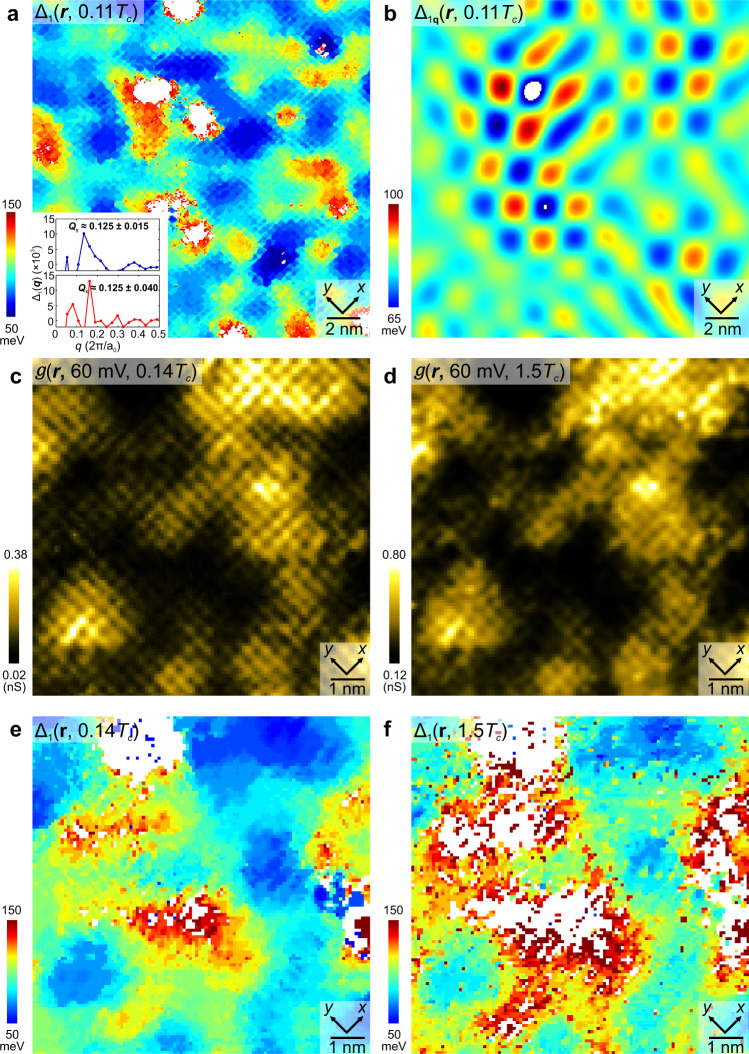
Energy-gap Modulations from Superconductive to Pseudogap Phase. **a** Measured ${\Delta }_{1}\left({{{{{\boldsymbol{r}}}}}}\right)$ within 20 nm × 20 nm FOV at *T* = $0.11{T}_{{{{{{\rm{c}}}}}}}$ = 4.2K. The energy-gap is measured from the energy of the coherence peak at *E* > 0. The inset shows the linecuts from ${{{{{\boldsymbol{q}}}}}}$ = (0, 0) to (0.5, 0)2π/*a*_0_ and from ${{{{{\boldsymbol{q}}}}}}$ = (0, 0) to (0, 0.5)2π/*a*_0_ in the measured ${\Delta }_{1}\left({{{{{\boldsymbol{q}}}}}}\right)$. The significant disorder as *q*→0 in is fitted to a cylindrical gaussian and subtracted. ${{{{{\boldsymbol{q}}}}}}\approx [(\pm 1/{{{{\mathrm{8,0}}}}});(0,\pm 1/8)]2{{{{{\rm{\pi }}}}}}/{a}_{0}$ peaks are present in both directions. The white areas represent regions where it is impossible to determine the coherence peak position ${\Delta }_{1}$. **b** Gap modulations ${\Delta }_{1q}\left({{{{{\boldsymbol{r}}}}}}\right)$ from 3(**a**). These are visualized at wavevectors ${{{{{\boldsymbol{q}}}}}}\approx [(\pm 1/{{{{\mathrm{8,0}}}}});(0,\pm 1/8)]2{{{{{\rm{\pi }}}}}}/{a}_{0}$ by Fourier filtering ${\Delta }_{1}\left({{{{{\boldsymbol{r}}}}}}\right)$ at the 1/8 peaks as shown in inset of 3a. The Gaussian filter size *σ*_*q*_ = 1.45 pixels (or equivalently 0.455 nm^−1^) in ***q***-space, which corresponds to 2.2 nm in ***r***-space. **c** Measured ${g}\left({{{{{\boldsymbol{r}}}}}},60{mV}\right)$ at *T* = 0.14${T}_{{{{{{\rm{c}}}}}}}$ = 5 K within 9.9 nm × 9.9 nm FOV. The $g\left({{{{{\boldsymbol{r}}}}}},60{mV}\right)$ manifests unidirectional charge modulations. **d** Measured $g\left({{{{{\boldsymbol{r}}}}}},60{mV}\right)$ at *T* = 1.5${T}_{{{{{{\rm{c}}}}}}}$ = 55 K in the identical FOV as (**c**). No change has been detected in $g\left({{{{{\boldsymbol{r}}}}}},60{mV}\right)$ at *T* = 55 K. **e** Measured ${\Delta }_{1}\left({{{{{\boldsymbol{r}}}}}}\right)$ at *T* = 0.14${T}_{{{{{{\rm{c}}}}}}}$ = 5 K shows the spatial variation of the coherence peak at *E* > 0. **f** Measured$\,{\Delta }_{1}\left({{{{{\boldsymbol{r}}}}}}\right)$ at *T* = 1.5${T}_{{{{{{\rm{c}}}}}}}$ = 55 K in the identical FOV as (**c**), (**d**) and (**e**). The spatial variation of the coherence peak is highly similar to (**e**).

At *T* = 4.2 K we first measure $g({{{{{\boldsymbol{r}}}}}},V)$ in a 20 nm-square FOV (see “Methods” section and Supplementary Fig. 5). The average differential conductance $g(V)$ is shown as a blue curve in Fig. 1d, where the energy of the coherence peak is determined from a local maximum in $g(V)$ for $V > 0$ (identified by a black vertical arrow). Measuring this energy versus location yields the so-called gapmap ${\Delta }_{1}\left({{{{{\boldsymbol{r}}}}}}\right)$ as shown in Fig. 3a. Fourier analysis of ${\Delta }_{1}\left({{{{{\boldsymbol{r}}}}}}\right)\,$yields ${\Delta }_{1}\left({{{{{\boldsymbol{q}}}}}}\right),$ which exhibits significant disorder as ${{{{{\boldsymbol{q}}}}}}\to {{{\bf{0}}}}$ (Supplementary Fig. 5b). But, by fitting the central peak to a cylindrical gaussian, and then subtracting it from ${\Delta }_{1}\left({{{{{\boldsymbol{q}}}}}}\right)$, we find four maxima at ${{{{{\boldsymbol{q}}}}}}\approx [(\pm 0.125\pm 0.040,0);(0,\pm 0.125\pm 0.015)]2{{\pi }}/{a}_{0}$ (inset in Fig. 3a). These are the energy-gap modulations with period approximately $8{a}_{0}$, that have been previously reported^35,37,38^ for samples with $p\approx 0.17$, and are the signature of a PDW state coexisting with *d*-symmetry superconductivity at low temperature. Fourier filtration of Fig. 3a retaining only modulations at ${{{{{\boldsymbol{q}}}}}}\approx \left[ \left(\pm 1/8,\,0\right); \left(0,\pm1/8\right)\right]2\pi /{a}_{0}$ yields an accurate image of the PDW gap modulations as seen in Fig. 3b. But when the same procedures are carried out at $T=1.5{T}_{{{{{{\rm{c}}}}}}}=55\,{{{{{\rm{K}}}}}}$, the coherence peaks from which the gap is defined have so diminished that an equivalent gapmap is difficult to achieve. For example, Fig. 3c, d show the measured $g({{{{{\boldsymbol{r}}}}}},60\,{mV})$ in an identical 10 nm-square FOV at $T=0.14{T}_{{{{{{\rm{c}}}}}}}=5\,{{{{{\rm{K}}}}}}$ and $T=1.5{T}_{{{{{{\rm{c}}}}}}}=55\,{{{{{\rm{K}}}}}}$. Cross correlation analysis of $g({{{{{\boldsymbol{r}}}}}},V)$ at *T* = $0.14{T}_{{{{{{\rm{c}}}}}}}$ and of $g({{{{{\boldsymbol{r}}}}}},V)$ at *T* = $1.5{T}_{{{{{{\rm{c}}}}}}}$ in this FOV versus bias voltage V, yield a normalized cross correlation coefficients around 0.9 for practically all energies (Supplementary Note 3), thus indicating that virtually no changes have occurred in spatial arrangements of electronic structure upon entering the PG phase. The major exception is in the energy range $+100\,{{{{{\rm{meV}}}}}} \, < \, E \, < \, +160\,{{{{{\rm{meV}}}}}}$ wherein the feature denoted coherence peak (arrow Fig. 1d) diminishes strongly in amplitude. This, however, makes comparison of the ${\Delta }_{1}\left({{{{{\boldsymbol{r}}}}}}\right)$ in same FOV at *T* = 5 K and *T* = 55 K challenging. Figure 3e, f shows the measured ${\Delta }_{1}\left({{{{{\boldsymbol{r}}}}}}\right)$ and, where it is possible to determine the energy, no changes have occurred in spatial arrangements of energy gaps either. The cross-correlation coefficient between the Δ_1_(***r***, 0.14*T*_C_) and Δ_1_(***r***, 1.5*T*_C_) is 0.685 indicating that the PDW state found at $T\ll {T}_{{{{{{\rm{c}}}}}}}$ remains robustly present at $1.5{T}_{{{{{{\rm{c}}}}}}}$ deep into the pseudogap phase.

### Temperature evolution in the QPI signature of a PDW state

**Fig. 4:**
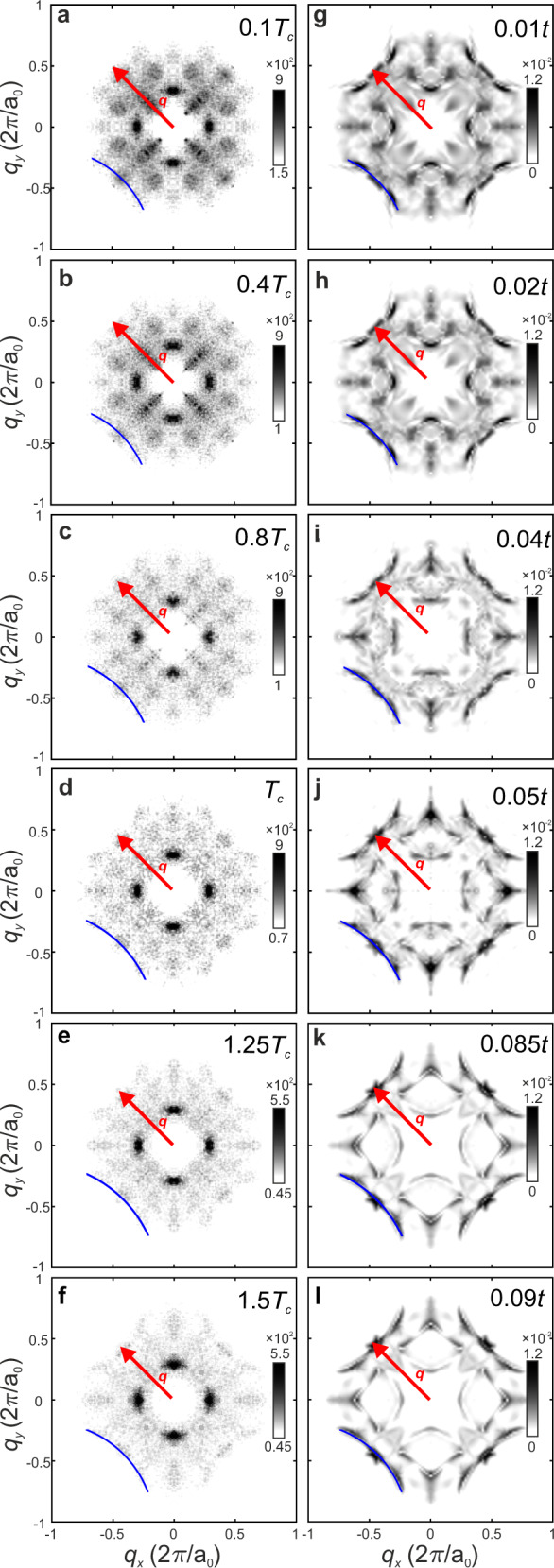
Temperature Dependence of QPI Signature of a PDW. **a**–**f**. Measured QPI signature $\Lambda ({{{{{\boldsymbol{q}}}}}},20\,{{{{{\rm{meV}}}}}})$ for Bi_2_Sr_2_CaDyCu_2_O_8_ (doping level *p* ≈ 0.08) at temperatures *T* = (**a**) 0.1*T*_c_, (**b**) 0.4*T*_c_, (**c**) 0.8*T*_c_, (**d**) *T*_c_, (**e**) 1.25*T*_c_, and (**f**) 1.5*T*_c_. **g**–**j**. Predicted QPI signature ${\Lambda }_{{{{{{\rm{P}}}}}}}({{{{{\boldsymbol{q}}}}}},20\,{{{{{\rm{meV}}}}}})$ of 8${a}_{0}$ PDW state that coexists with DSC state at temperatures *T* = (**g**) 0.01*t*, (**h**) 0.02*t*, (**i**) 0.04*t*, and (**j**) 0.05*t*. Theoretically, it is assumed that the short-range discommensurate nature of the charge order, as seen in the experiments^56^, will lead to reduced intensity of the density wave Bragg peaks compared to the long-range PDW driven charge order obtained in our mean-field analysis. Accordingly, the non-dispersing charge order Bragg peaks at wavevectors ***q*** = ±*n****Q***_p_, *n* = 0, 1, 2, …,7, in PDW+DSC state and ***q*** = ±n(2***Q***_p_), *n* = 0, 1, 2, 3, in the pure PDW state are suppressed by a factor of 100 in ${\Lambda }_{{{{{{\rm{P}}}}}}}\left({{{{{\boldsymbol{q}}}}}},20\,{{{{{\rm{meV}}}}}}\right)$, which helps in highlighting much weaker wavevectors emerging from impurity scattering. ${\Lambda }_{{{{{{\rm{P}}}}}}}\left({{{{{\boldsymbol{q}}}}}},20\,{{{{{\rm{meV}}}}}}\right)$ is computed for unidirectional PDW in a 56×56 lattice and symmetrized for plotting. Features at ***q*** ≈ (±1/4, ±1/4)2π/*a*_0_ extending in nodal directions are labeled by a red arrow. **k**–**l**. Predicted ${\Lambda }_{{{{{{\rm{P}}}}}}}({{{{{\boldsymbol{q}}}}}}, 20\,{{{{{\rm{ meV}}}}}})$ of pure 8${a}_{0}$ PDW state at temperatures *T* = (**k**) 0.085*t* and (**l**) 0.09*t*. Measured $\Lambda ({{{{{\boldsymbol{q}}}}}},20\,{{{{{\rm{meV}}}}}})$ in (**a**–**f**) for *T* = 0.1*T*_c_ ~ 1.5*T*_c_ are in good agreement with the simulation results in (**g**–**l**). The length of the arc-like feature (indicated by blue curves) subtending (±1, ±1)$2{{{{{\rm{\pi }}}}}}/{a}_{0}$ increases from PDW+DSC to pure PDW state, which is a key feature of charge order driven by PDW. The intensity of $\Lambda ({{{{{\boldsymbol{q}}}}}},20\,{{{{{\rm{meV}}}}}})$ and ${\Lambda }_{{{{{{\rm{P}}}}}}}\left({{{{{\boldsymbol{q}}}}}},20\,{{{{{\rm{meV}}}}}}\right)$ decreases as the temperature increases.

Next, we measure $g({{{{{\boldsymbol{r}}}}}},V,T)$ for $-34\,{{{{{\rm{mV}}}}}} < V < 34\,{{{{{\rm{mV}}}}}}$ at a sequence of temperatures ${0.1T}_{{{{{{\rm{c}}}}}}}\le T\le 1.5{T}_{{{{{{\rm{c}}}}}}}$. Then $Z\left({{{{{\boldsymbol{r}}}}}},V\right)=g({{{{{\boldsymbol{r}}}}}},+V)/g({{{{{\boldsymbol{r}}}}}},-V)$ is evaluated for each temperature, and the power-spectral-density Fourier transforms $Z\left({{{{{\boldsymbol{q}}}}}},V\right)$ are derived. Hence, $\Lambda ({{{{{\boldsymbol{q}}}}}},{\Delta }_{0})={\sum }_{E\cong 0}^{{\Delta }_{0}}Z({{{{{\boldsymbol{q}}}}}},E)$ is calculated at each temperature where ${\Delta }_{0}=20\,{{{{{\rm{meV}}}}}}\,$is the observed energy above which dispersive scattering interference is no longer detectable^10,35^. The measured temperature dependence of $\Lambda \left({{{{{\boldsymbol{q}}}}}},{\Delta }_{0}\right)$ for ${0.1T}_{{{{{{\rm{c}}}}}}}\le T\le 1.5{T}_{{{{{{\rm{c}}}}}}}$ is shown in the left column in Fig. 4. The initial $\Lambda \left({{{{{\boldsymbol{q}}}}}},{\Delta }_{0}\right)$ features at *T*=4.2K are exactly as expected from theory and as observed by experiment at *p* = 0.17, for a PDW coexisting with a *d*-wave superconductor^35^. As temperature increases the characteristics remain strikingly unchanged except that the intensity become significantly weaker. That the passage through *T*_c_ exhibits almost no signature in $\Lambda \left({{{{{\boldsymbol{q}}}}}},{\Delta }_{0}\right)$, is unexpected if the scattering interference in Λ$\left({{{{{\boldsymbol{q}}}}}},{\Delta }_{0}\right)$ is only due to the *d*-wave superconductivity. If however, a PDW state exists both below and above *T*_c_ this is what might logically be expected. Moreover, quantitative theoretical predictions for Λ$\left({{{{{\boldsymbol{q}}}}}},{\Delta }_{0}\right)$ for a $\lambda =8{a}_{0}$ PDW using the RMFT model, predict $Z\left({{{{{\boldsymbol{q}}}}}},E\right)\,$ surrounding a point-like scatterer and hence ${\Lambda }_{{{{{{\rm{P}}}}}}}\left({{{{{\boldsymbol{q}}}}}},{\Delta }_{0}\right)={\sum }_{E\cong 0}^{{\Delta }_{0}}Z({{{{{\boldsymbol{q}}}}}},E)$ (Supplementary Note 5). Comparing our Λ$\left({{{{{\boldsymbol{q}}}}}},{\Delta }_{0}\right)$ data to the RMFT-derived predictions ${\Lambda }_{{{{{{\rm{P}}}}}}}\left({{{{{\boldsymbol{q}}}}}},{\Delta }_{0}\right)$ for a $\lambda =8{a}_{0}$ PDW in the right column in Fig. 4 shows how the key features in the experiments are reproduced in the theory over the whole range of temperatures. Features at ${{{{{\boldsymbol{q}}}}}}\approx (\pm 1/4,\,\pm 1/4)2\pi /{a}_{0}$ extending in nodal directions disappear with the transition from superconducting state to pseudogap; this is reproduced in our theory as a consequence of vanishing DSC component (indicated by red arrow in Fig. 4 and Supplementary Fig. 12). Further, the measured length of the $\Lambda \left({{{{{\boldsymbol{q}}}}}},{\Delta }_{0}\right)\,$arc features about $(\pm 1,\,\pm 1)2\pi /{a}_{0}$ increase continuously from superconducting to pseudogap phase (Supplementary Note 6). This temperature evolution of the arc length in ${\Lambda }_{{{{{{\rm{P}}}}}}}({{{{{\boldsymbol{q}}}}}},20\,{{{{{\rm{meV}}}}}})$ from PDW+DSC state to pure PDW state in the RMFT model, has indistinguishable characteristics (Supplementary Fig. 14). Moreover, superimposing the experimental and theoretical maps shows nearly identical positioning of dominant QPI features in q-space (Supplementary Fig. 12). The implication is that the PDW state which definitely exists at lowest temperatures^35–38^, continues to exist into pseudogap phase. But in that case, since that pseudogap is often (but not always) reported to support no supercurrents, it would have to be in a strongly phase fluctuating PDW phase^32,33,49–53^.

### Comparison of QPI Signature of a CDW and PDW State

**Fig. 5:**
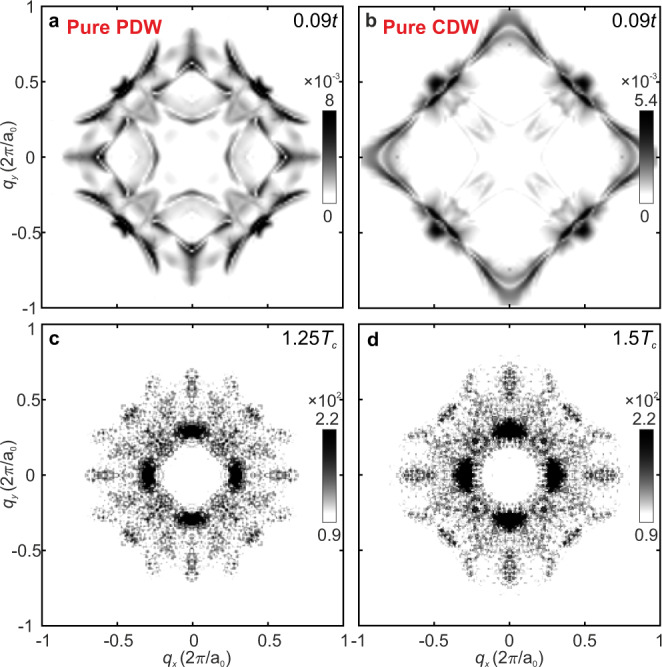
Discrimination of CDW from PDW QPI Signature in the Pseudogap State. **a** Predicted QPI signature ${\Lambda }_{{{{{{\rm{P}}}}}}}({{{{{\boldsymbol{q}}}}}},20\,{{{{{\rm{meV}}}}}})$ of pure 8${a}_{0}$ PDW state at *T* = 0.09*t*. **b** Predicted ${\Lambda }_{{{{{{\rm{C}}}}}}}({{{{{\boldsymbol{q}}}}}},20\,{{{{{\rm{meV}}}}}})$ of pure 4${a}_{0}$ CDW state at *T* = 0.09*t*. The CDW states show very different features compared to the PDW state. **c** Measured $\Lambda ({{{{{\boldsymbol{q}}}}}},20\,{{{{{\rm{meV}}}}}})$ of Bi_2_Sr_2_CaDyCu_2_O_8_ (*p* ≈ 0.08) for the pseudogap phase at *T* = 1.25*T*_c_. **d** Measured $\Lambda ({{{{{\boldsymbol{q}}}}}},20\,{{{{{\rm{meV}}}}}})$ of Bi_2_Sr_2_CaDyCu_2_O_8_ (*p* ≈ 0.08) for the pseudogap phase at *T* = 1.5*T*_c_. **e** The measurements of the pseudogap phase agree much better with the pure PDW scenario (**a**) than with the pure CDW (**b**).

Finally, we consider the widely promulgated hypothesis^15–17^, that the pseudogap phase is a primary CDW state, whose charge density modulation breaks the translational symmetry of the cuprate pseudogap phase. First, we note the very sharp distinction between these states: the mean-field order parameter of a PDW at wavevector ${{{{{{\boldsymbol{Q}}}}}}}_{{{{{{\rm{P}}}}}}}$ is $\langle {c}_{{{\boldsymbol{k}}},\uparrow }^{{{\dagger}} }{c}_{-{{\boldsymbol{k}}}+{{{\boldsymbol{Q}}}}_{{{{{{\rm{P}}}}}}},\downarrow }^{{{\dagger}} }\rangle$, whereas for a CDW at wavevector ${{{{\boldsymbol{Q}}}}}_{{{{\rm{C}}}}}$ it is ${\sum }_{\sigma }\langle {c}_{{{\boldsymbol{k}}},\sigma }^{{{\dagger}} }{c}_{{{\boldsymbol{k}}}+{{{\boldsymbol{Q}}}}_{{{{{{\rm{C}}}}}} },\sigma}\rangle$. Second, while a periodically modulating energy gap is a key PDW signature (Fig. 3a), ***r***-space energy gap modulation should be weak in a CDW state, where it is charge density which modulates. Third, the quasiparticles and their scattering interference are highly distinct for the two states. A primary CDW order by itself does not exist as a stable self-consistent solution of the RMFT *t-J* model at any temperatures or dopings that we have considered. However, we can study STM signatures of the CDW order non-self-consistently. Figure 5a shows the predicted Λ_P_$\left({{{{{\boldsymbol{q}}}}}},{\Delta }_{0}\right)={\sum }_{E\cong 0}^{{\Delta }_{0}}Z({{{{{\boldsymbol{q}}}}}},E)$ for a $\lambda =8{a}_{0}$ PDW in the CuO_2_ pseudogap phase, while Fig. 5b shows the equivalent predictions for a $\lambda =4{a}_{0}$ CDW (Supplementary Note 5). In Fig. 5c, d we show the measured $\Lambda \left({{{{{\boldsymbol{q}}}}}},{\Delta }_{0}\right)\,$at $T=1.25{T}_{c}$ and $T=1.5{T}_{c}$ ($\Lambda \left({{{{{\boldsymbol{q}}}}}},{\Delta }_{0}\right)\,$analysis details are discussed in Supplementary Note 6). Clearly, the measured $\Lambda \left({{{{{\boldsymbol{q}}}}}},{\Delta }_{0}\right)$ is in superior agreement with the ${\Lambda }_{{{{{{\rm{P}}}}}}}\left({{{{{\boldsymbol{q}}}}}},{\Delta }_{0}\right)$ signature of a $\lambda =8{a}_{0}$ PDW rather than with that of a $\lambda =4{a}_{0}$ CDW. The energy evolution of the wavevectors is visualized in the measured $Z\left({{{\boldsymbol{q}}}},V,55\,{{{\rm{K}}}}\right)$ from 4 mV to 20 mV (movie S2 and Supplementary Note 7). The wavevectors evolve dispersively with energy only by a small amount. Finally, The $\Lambda \left({{{{{\boldsymbol{q}}}}}},{\Delta }_{0}\right)$ in the pseudogap phase forms an open contour near the lines (±1, 0)$2{{\pi }}/{a}_{0}$ and (0, ±1)$2{{\pi }}/{a}_{0}$; this is consistent with the open contours in the ${\Lambda }_{{{{{{\rm{P}}}}}}}\left({{{{{\boldsymbol{q}}}}}},{\Delta }_{0}\right)$ signature of a $\lambda =8{a}_{0}$ PDW but distinct from the closed contours of a $\lambda =4{a}_{0}$ CDW. Therefore, predictions of a pure PDW theory correspond well and in detail to the complex patterns of the quasiparticle scattering that are actually observed in the pseudogap phase of Bi_2_Sr_2_CaDyCu_2_O_8_.

To summarize: strongly underdoped Bi_2_Sr_2_CaDyCu_2_O_8_ at *p*~0.08 and *T* = 5 K exhibits the 8*a*_0_-periodic ${\Delta }_{1}({{{\boldsymbol{r}}}})$ modulations characteristics of a PDW coexisting with superconductivity^35,37,38^ (Figs. 2d and 3b). Increasing temperature from the superconducting into the pseudogap phase, seems to retain these real-space phenomena apparently thermally broadened but otherwise unchanged (Fig. 3c–f). More obviously, the measured scattering interference signature^10^ $\Lambda ({{{\boldsymbol{q}}}})$ evolves from correspondence with ${\Lambda }_{{{{\rm{P}}}}}({{{\boldsymbol{q}}}})$ predicted for an 8*a*_0_-periodic PDW coexisting with superconductivity^35^ into that predicted for a pure 8*a*_0_-periodic PDW above the superconductive *T*_*c*_ in the pseudogap phase (Fig. 4). Furthermore, this signature is highly distinct from $\Lambda_{{{{{{\rm{C}}}}}}} ({{{{{\boldsymbol{q}}}}}})$ predicted for a 4*a*_0_-periodic CDW (Fig. 5). The clear inference from all these observations is that the Bi_2_Sr_2_CaDyCu_2_O_8_ pseudogap phase contains a PDW state, whose quantum phase is fluctuation dominated.

## Methods

Single crystals of Bi_2_Sr_2_CaDyCu_2_O_8_ with hole doping level of *p* ≈ 8% and *T*_c_ = 37 ± 3 K were synthesized using the floating zone method with doping controlled by oxygen depletion. The samples were cleaved in cryogenic ultrahigh vacuum at *T* = 4.2 K to reveal an atomically flat BiO surface, and then inserted into STM. All measurements are carried out using tungsten tips in a variable temperature (the range is *T* = 4.2–55 K) spectroscopic imaging STM system with thermal fluctuations less than 1 mK. The PG gap map ${{{{{{\boldsymbol{\Delta }}}}}}}_{1}\left({{{{{\boldsymbol{r}}}}}}\right)$ were measured with the resolution of 128 pixels × 128 pixels. The experimental parameters include setpoint voltage 800 mV, setpoint current 800 pA, bias voltage ${V}_{B}$ = −800 mV– 800 mV and 161 discrete energy layers. The QPI images were measured with the resolution of 256 pixels × 256 pixels. The experimental parameters of the QPI measurements include spectroscopic setpoint voltage 200 mV, setpoint current 200 pA, bias voltage ${V}_{B}$ = −34 mV – 34 mV and 35 discrete energy layers. The topography $T({{{{{\boldsymbol{r}}}}}})$ of the six temperatures studied in this paper are shown in Supplementary Figure 4. The presented QPI patterns were symmetrized to reduce the noise. In the QPI pattern, a circle with a locus located at ***q*** = **0** and a radius of 25 pixels is fitted to 2D Gaussian function and then removed.

## Supplementary information

Supplementary Information

Description of Additional Supplementary Files

Supplementary Movie 1

Supplementary Movie 2

## Data Availability

All data are available in the main text, in the Supplementary Information and on Zenodo^54^. Additional information is available from the corresponding author upon reasonable request.

## Code availability

The data analysis codes used in this study are available from the corresponding author upon reasonable request.
