# Supplementary material for: Scattering interference signature of a pair density wave state in the cuprate pseudogap phase
Source: Nat Commun. 2021 Oct 19;12:6087. doi: 10.1038/s41467-021-26028-x (PMC8526682; doi:10.1038/s41467-021-26028-x)
Supplement: Supplementary file 1 — Supplementary Information [file 41467_2021_26028_MOESM1_ESM.pdf]

Supplementary Information for

Scattering Interference Signature of a Pair Density Wave State in the Cuprate Pseudogap Phase

Shuqiu Wang, Peayush Choubey, Yi Xue Chong, Weijiong Chen, Wangping Ren,

H. Eisaki, S. Uchida, P.J. Hirschfeld and J.C. Séamus Davis

Supplementary Note 1

Renormalized mean-field theory of the extended t-J model

The extended *t-J* model on a square lattice is given by

$$H = -\sum_{(i,j),\sigma} P_G t_{ij}\left(c_{i\sigma}^{\dagger}c_{j\sigma} + h.c.\right)P_G + J\sum_{<i,j>}\boldsymbol{S}_i \cdot \boldsymbol{S}_j, \tag{1}$$

where $c_{i\sigma}^{\dagger}$ creates an electron at the lattice site i with spin σ. The hopping amplitude t_{ij} is taken to be t and t' when i, j are the nearest-neighbor (NN) and next-nearest-neighbor (NNN) sites, respectively. $< i, j >$ and (i, j) denotes only NN, and both NN and NNN sites, respectively. The Gutzwiller projector P_G projects out all configurations with doubly occupied sites from Hilbert space. Finally, $\boldsymbol{S}_i$ represents the spin operator at site i, and J is the superexchange coupling between spins residing at NN sites. The no-double-occupancy constraint can be implemented by employing the Gutzwiller approximation, in which the projection operator P_G is replaced by site-dependent Gutzwiller renormalization factors g^t and g^s for hoppings and superexchange coupling, respectively. The resulting renormalized Hamiltonian now reads,

$$H = -\sum_{(i,j),\sigma} g_{ij}^{t} t_{ij}\left(c_{i\sigma}^{\dagger}c_{j\sigma} + h.c.\right) + J\sum_{<i,j>}[g_{ij}^{S,Z} S_i^Z S_j^Z + g_{ij}^{S,xy}(\frac{S_i^+ S_j^- + S_i^- S_j^+}{2})] \tag{2}$$

Further progress can be made by mean-field decoupling of the renormalized Hamiltonian in density and pairing channels with ensuing mean-fields hole density δ_i, bond-field $\chi_{ij\sigma}$, magnetic moment m_i, and pair potential $\Delta_{ij\sigma}$ defined as

$$\Delta_{ij\sigma} = \sigma < \Psi_0 | c_{i\sigma} c_{j\bar{\sigma}} | \Psi_0 >, \tag{3}$$

$$\chi_{ij\sigma} = < \Psi_0 | c_{i\sigma}^{\dagger} c_{j\sigma} | \Psi_0 >, \tag{4}$$

$$\delta_i = 1 - < \Psi_0 | \textstyle\sum_\sigma n_{i\sigma} | \Psi_0 >, \tag{5}$$

$$m_i = < \Psi_0 | S_i^Z | \Psi_0 >, \tag{6}$$

where, $|\Psi_0>$ is the unprojected ground state wavefunction. A direct diagonalization of the resulting mean-field Hamiltonian will not yield the lowest energy state, however, as the Gutzwiller factor themselves depend on the local mean-fields. Instead, the ground state energy $E_g =< \Psi_0|H|\Psi_0 >$ has to be minimized with respect to $|\Psi_0 >$ under constraints that the total electron density is fixed and $|\Psi_0 >$ is normalized[1]. This leads to following renormalized mean-field Hamiltonian for paramagnetic states (m_i = 0).

$$H_{MF} = \sum_{(i,j),\sigma} \epsilon_{ij\sigma} c_{i\sigma}^{\dagger} c_{j\sigma} + h.c. + \sum_{<i,j>,\sigma} \sigma D_{ij\sigma}^{*} c_{i\sigma} c_{j\bar{\sigma}} + h.c. - \sum_{i,\sigma} \mu_{i\sigma} n_{i\sigma}, \quad (7)$$

where,

$$\epsilon_{ij\sigma} = -g_{ij}^{t} t_{ij} - \delta_{ij,<ij>} \frac{3}{4} J g_{ij}^{S} \chi_{ij\sigma}^{*} \quad (8)$$

$$D_{ij\sigma} = -\delta_{ij,<ij>} \frac{3}{4} J g_{ij}^{S} \Delta_{ij\sigma} \quad (9)$$

$$\mu_{i\sigma} = \mu + \frac{3}{4} J \sum_{j\sigma'} (|\Delta_{ij\sigma'}|^2 + |\chi_{ij\sigma'}|^2) \frac{dg_{ij}^{S}}{dn_{i\sigma}} + t_{ij} \sum_{j\sigma'} (\chi_{ij\sigma'} + \chi_{ij\sigma'}^{*}) \frac{dg_{ij}^{t}}{dn_{i\sigma}} \quad (10)$$

Here, $\delta_{ij,<ij>}$ = 1 for NN sites and 0 otherwise. In this work, we have focused only on paramagnetic states since we are interested in charge ordering without any long-range spin ordering as very few experiments suggested the presence of any long-range magnetic order coexisting with charge order in $Bi_2Sr_2CaCu_2O_{8+\delta}$. In this scenario, the Gutzwiller renormalization factors are simply given by the following expressions[1]

$$g_{ij\sigma}^{t} = g_{ij}^{t} = g_i^{t} g_j^{t}; \; g_i^{t} = \sqrt{\frac{2\delta_i}{1-\delta_i}} \quad (11)$$

$$g_{ij}^{s,z} = g_{ij}^{s,xy} = g_{ij}^{s} = g_i^{s} g_j^{s}; \; g_i^{s} = \frac{2}{1+\delta_i} \quad (12)$$

Here, we have assumed that the above expressions are valid at all temperatures of interest[2]. In other words, we have approximated $T \neq 0$ Gutzwiller factors by their values at $T = 0$. Temperature effects enter the calculations via Fermi functions used in the evaluation of the mean-fields [Supplementary Eq. (3-6)]). The renormalized mean-field Hamiltonian in Supplementary Eq. (7) can be diagonalized by using a spin-generalized Bogoliubov transformation, yielding the following Bogoliubov-de Gennes (BdG) equation

$$\sum_j \begin{pmatrix} \epsilon_{ij\uparrow} & D_{ij\uparrow} \\ D_{ji\uparrow}^{*} & -\epsilon_{ij\downarrow} \end{pmatrix} \begin{pmatrix} u_j^n \\ v_j^n \end{pmatrix} = E_n \begin{pmatrix} u_i^n \\ v_i^n \end{pmatrix}. \quad (13)$$

The BdG equation has to be solved self-consistently as the matrix elements depend on the mean-fields, which, in turn, depend on the eigenvalues (u_i^n, v_i^n) and eigenvectors E_n. The paramagnetic ground state of the *t-J* model treated within aforementioned renormalized mean-field theory (RMFT) is a uniform d-wave superconductor (DSC). However, we are interested in pair density wave (PDW) solutions which have been shown to be very close in energy to the DSC state within RMFT[1-3] as well as in more rigorous numerical schemes like variational Monte-Carlo[4,5] and tensor networks[6]. PDW states can be obtained by

initializing BdG equation [Supplementary Eq. (13)] with modulating pair-field (keeping other mean-fields uniform) with the following form.

$$\Delta_{i,i+\hat{x}} = \Delta_0 + \Delta_Q \cos(\boldsymbol{Q}_P \cdot \mathbf{R}_i) \tag{14}$$

$$\Delta_{i,i+\hat{y}} = -\Delta_0 - \Delta_Q \cos[\boldsymbol{Q}_P \cdot \left(\mathbf{R}_i - \frac{a_0}{2}\hat{n}\right)] \tag{15}$$

Here, the modulation wavevector is chosen to be $\boldsymbol{Q}_P = (\pm 1/8, 0)\frac{2\pi}{a_0}$ based on experimental evidences[7,8,9]. A bond-centered PDW state with coexisting d-wave superconductivity (PDW+DSC) can be obtained as a self-consistent solution using a finite $\Delta_0 < \Delta_Q$, whereas a pure PDW state can be obtained by setting $\Delta_0 = 0$ in the initial seed. We note that a computationally more efficient scheme to study unidirectional modulating states (in absence of disorder) is obtained by exploiting translational invariance in direction orthogonal to modulations. Here, BdG equations on 2D lattice are Fourier transformed in the orthogonal direction to yield quasi-1D BdG equations. Details of this scheme can be found in Ref. [3]. Fig. 2(a), (c)-(f) in the main-text have been obtained using this scheme.

Results presented in the main-text were obtained using the parameter set $t = 400$ meV, $t' = -0.3t$, $J = 0.3t$. Further, we chose hole-doping $p = 0.125$, which is larger than the doping $p = 0.08$ at which experiments discussed in the main-text were performed because of the following reasons. First, it has long been known that the *t-J* model overestimates the doping scale of DSC dome by almost a factor of two. Experiments find the DSC dome to be in hole doping range $p \sim 0.05$-0.3 whereas in the RMFT *t-J* model (with the aforementioned parameter set) it turns out to be in the range 0.01-0.45 (at $T = 0$)[3]. If we account for this scale difference, then $p = 0.125$ will be closer to the experimental doping of $p = 0.08$. Second, it's hard to get converged PDW solutions at very low dopings as the derivatives of Gutzwiller factors, entering in the on-site potentials [Supplementary Eq. (10)], fluctuate strongly even with a small change in local doping[10]. This is more severe when solving the impurity problem. Finally, our conclusions mainly depend on just one premise: the low-temperature state is PDW+DSC and the high-temperature state is pure PDW, which does not depend on the actual doping level as long as it remains below a critical level ($p \sim 0.18$ at $T = 0$) to realize these states.

For the aforementioned parameter set, a self-consistent pure PDW state is obtained in temperature range $0 < T < 0.11t$ whereas the PDW+DSC state is found as a stable solution for $0 < T < 0.085t$. Both PDW and PDW+DSC states have almost equal energy per site, which is a few meV larger than the uniform DSC state[1,2,3,11]. This tiny energy difference between PDW+DSC and DSC state can be overcome by a variety of means, such as disorder which is not accounted for in the calculation. We have effectively assumed such effects to be present, leading to the PDW+DSC state at low-temperatures (in the range $0 < T < 0.085t$) and the pure PDW state at higher temperatures (in the range $0.085t < T < 0.11t$). In the PDW+DSC state, increasing temperature leads to a sharp

decrease in the uniform DSC component ($\Delta(\boldsymbol{q}=\mathbf{0})$) as shown in the main-text Figure 2e. For $0.05t < T < 0.085t$, $\Delta(\boldsymbol{q}=\mathbf{0})$ is very small but finite. This 'fragile PDW+DSC' state is a stable solution of the RMFT equations and not a computational artefact. This result is verified by the observation that lowering the self-consistency tolerance by an order of magnitude yields the same state.

In the main-text Fig. 2c and 2d, we showed spatial variation of hole density and d-wave gap order parameter, respectively. To complete the discussion of mean-fields, Supplementary Figure 2a-c show the spatial variation of NN bond mean-field χ_{ij} in PDW+DSC (at $T = 0.01t, 0.04t$) and pure PDW state (at $T = 0.09t$). We find that the modulations in χ_{ij} are typically of the size ~0.1t in PDW+DSC at low temperatures and ~0.05t in pure PDW states at higher temperatures. The bare bond fields are not physical observables, however. The physical expectation value of the bond operator ($c^{\dagger}_{i\sigma}c_{j\sigma}$) in the Gutzwiller projected state is the bond mean-field scaled by the Gutzwiller hopping factor: $\chi^{o}_{ij} = g^{t}_{ij}\chi_{ij}$ [12]. We can define the NN bond order at a given lattice site *i* as $\chi^{o}_{i} = (\chi^{o}_{i,i+\hat{x}} + \chi^{o}_{i,i-\hat{x}} + \chi^{o}_{i,i+\hat{y}} + \chi^{o}_{i,i-\hat{y}})/4$, where $i \pm \hat{x}\,(\hat{y})$ represent NN sites to *i* along x(y)-direction. As evident from Supplementary Figure 2d, the size of modulations in the bond order turns out to be an order of magnitude smaller than the bare mean-field. Similar to the case of hole density, the reduction in the modulation amplitude of bond variables for higher temperatures is a consequence of the reduction in the PDW gap order parameter (Fig. 2e in the main-text). Finally, we note that the bond order in both PDW and PDW+DSC states has a dominant *d*-form factor[11].

In order to compute local density of states (LDOS), we first obtain lattice Green's functions $G_{ij}(E)$ using the eigenvalues and eigenvectors of the BdG matrix [Supplementary Eq. (13)].

$$G_{ij}(E) = g^{t}_{ij}\sum_{n}\frac{u^{n}_{i}u^{n*}_{j}}{\omega - E_{n} + i0^{+}} \tag{16}$$

Here, 0^{+} is a small artificial broadening set to be $0.01t$, and the sum runs over all the eigenvalues. The diagonal lattice Green's function yields total LDOS at a site:

$$N_{i}(E) = -\frac{2}{\pi}Im[G_{ii}(E)], \tag{17}$$

where, Im represents imaginary part and the factor 2 accounts for spin degeneracy. Differential conductance measured in an STM experiment is, however, proportional to the sample's LDOS evaluated at the STM tip position[13]. Thus, we must compute the continuum LDOS few angstroms above the exposed BiO layer in $Bi_2Sr_2CaCu_2O_{8+\delta}$ for a meaningful comparison with the experimental data. Accordingly, we obtain continuum Green's function $G(\boldsymbol{r},\boldsymbol{r}';E)$ via a basis transformation[14] from lattice to continuum space where the matrix elements of the transformation are given by the Wannier function $W_{i}(\boldsymbol{r})$ centered at lattice site *i*.

$$G(\boldsymbol{r},\boldsymbol{r}';E) = \sum_{ij} W_i(\boldsymbol{r})G_{ij}(E)W_j^*(\boldsymbol{r}') \quad (18)$$

The imaginary part of the diagonal continuum Green's function yields LDOS at a continuum point $\boldsymbol{r}$.

$$N(\boldsymbol{r},E) = -\frac{2}{\pi} Im[G(\boldsymbol{r},\boldsymbol{r};E)] \quad (19)$$

We have obtained the continuum LDOS at a height ~4Å above the BiO layer in $Bi_2Sr_2CaCu_2O_{8+\delta}$ employing a first-principles Cu-3d_{x2-y2} Wannier function obtained using the Wannier90 package, identical to that used in Ref. [3,15] and very similar to that in Ref. [16].

Supplementary Fig. 1a shows the continuum LDOS map at $E = \Delta_1$ in the pure PDW state at $T = 0.09t$. The LDOS shows a periodicity of $4a_0$. Supplementary Fig. 1b shows spectra at eight Cu positions marked in the panel 1a. Sharp features present at higher energies are expected to be broadened by inelastic scattering, which has been shown in Ref. [17] to be essential to account for the spectral lineshapes in underdoped cuprates. In that work, it was shown that the effects of inelastic scattering can be simply incorporated by adding a linear-in-energy term $i\Gamma = i\alpha|E|$ to the constant artificial broadening $i0^+$ used in calculation of lattice Green's function [Supplementary Eq.(16)]. Using the experimental fits presented in Ref. [17], we set $\alpha = 0.25$. Supplementary Fig. 1c shows the continuum LDOS incorporating the linear inelastic scattering. All LDOS, $Z(\boldsymbol{q},E)$, and $\Lambda_P(\boldsymbol{q},\Delta_0)$ results presented in the main-text, and gap map results presented in Supplementary Figure 7 have been obtained after accounting for the inelastic scattering.

We note that a finite value of artificial broadening $i0^+$ used in our calculations is responsible for non-zero LDOS at zero bias in the PDW+DSC state, as seen in Fig. 2a. Indeed, with decreasing artificial broadening, the zero-bias LDOS in PDW+DSC state approaches 0 due to presence of nodes in the quasiparticle spectrum[18], as evident from Supplementary Figure 3. On the contrary, the zero-bias LDOS saturates at a finite value in pure PDW state due to the presence of Bogoliubov-Fermi surface[1,18].

Supplementary Note 2

PG gap $\Delta_1(\boldsymbol{r})$ modulation detection

We determine the gap map $\Delta_1(\boldsymbol{r})$ by measuring the energy of the coherence peak in each dI/dV spectrum at $E > 0$. Supplementary Figure 5b shows the magnitude of the power-spectral-density Fourier transform $\Delta_1(\boldsymbol{q})$ of the gap map $\Delta_1(\boldsymbol{r})$ in Figure 5a. There is strong disorder in $\Delta_1(\boldsymbol{q})$ surrounding $\boldsymbol{q} = \boldsymbol{0}$. The feature at a length of 1/5 in the (0, 0)-(1, 1) direction is related to the BiO supermodulation. The feature at about 20 degrees off the (0, 0)-(1, 0) direction at a length about 1/6 is possibly related to the electronic disorder. In Supplementary Figure 5, we show $\Delta_1(\boldsymbol{q})$ intensities before and after the

exponential background has been subtracted. After the background is subtracted, the maxima at $\boldsymbol{Q}_P \approx (0, \pm 1/8)2\pi/a_0$ and $\boldsymbol{Q}_P \approx (\pm 1/8, 0)2\pi/a_0$ become clearly visible. This analysis provides one type of experimental evidence of the $8a_0$ modulations in $\Delta_1(\boldsymbol{r})$.

We apply a computationally two-dimensional lock-in technique to obtain the amplitude $\Delta_{q_i}(\boldsymbol{r})$ of the gap modulation $\Delta_1(\boldsymbol{r})$ at $\boldsymbol{q}_i$. $\Delta_1(\boldsymbol{r})$ is multiplied by $e^{i\boldsymbol{q}_i \cdot \boldsymbol{r}}$ and integrated over a Gaussian filter to obtain the complex-values lock-in signal[9,19]

$$\Delta_{q_i}(\boldsymbol{r}) = \frac{1}{\sqrt{2\pi}\sigma} \int d\boldsymbol{R} \Delta_1(\boldsymbol{R}) e^{i\boldsymbol{q}_i \cdot \boldsymbol{R}} e^{\frac{|\boldsymbol{r}-\boldsymbol{R}|^2}{2\sigma^2}} \tag{20}$$

Where $\boldsymbol{q}$ denotes the wavevector of interest and σ the average length-scale in $\boldsymbol{r}$-space. This technique is implemented in $\boldsymbol{q}$-space

$$\Delta_{q_i}(\boldsymbol{r}) = \mathcal{F}^{-1} \Delta_{q_i}(\boldsymbol{q}) = \mathcal{F}^{-1}[\mathcal{F}(\Delta_1(\boldsymbol{r}) e^{i\boldsymbol{q}_i \cdot \boldsymbol{r}}) \cdot \frac{1}{\sqrt{2\pi}\sigma_q} e^{-\frac{q^2}{2\sigma_q{}^2}}] \tag{21}$$

where $\sigma_q = 1/\sigma$ is the cut-off length in $\boldsymbol{q}$-space. σ is specified to capture only the relevant image distortions.

Supplementary Note 3

Atomic precision image registration

In the temperature dependence experiments, $T(\boldsymbol{r}, 5\text{ K})$ and $T(\boldsymbol{r}, 55\text{ K})$ are measured in the same field of view with sub-unit-cell resolution. The data are processed by performing the Lawler-Fujita procedure[20] that maps the data onto a perfectly periodic lattice without lattice distortions. The data are subsequently corrected using shear transformation to maintain the C4 symmetry of the CuO_2 crystal lattice. After the topographs are corrected, $T(\boldsymbol{r}, 5\text{ K})$ and $T(\boldsymbol{r}, 55\text{ K})$ are registered to the exact same FOV with atom-by-atom precision as shown in Supplementary Figure 6a and b. Subtraction of $T(\boldsymbol{r}, 5\text{ K})$ from $T(\boldsymbol{r}, 55\text{ K})$ gives rise to $\delta T(\boldsymbol{r})$ in Supplementary Figure 6c. The differences between $T(\boldsymbol{r}, 5\text{ K})$ and $T(\boldsymbol{r}, 55\text{ K})$ are noise and distortions in individual unit cells. They are not relevant to the demonstration from $\delta T(\boldsymbol{r})$ that the FOVs of 5 K and 55 K are identical.

The differential conductance map $g(\boldsymbol{r}, V)$ is simultaneously acquired with $T(\boldsymbol{r})$. Applying the same image processing procedures of correcting $T(\boldsymbol{r})$ to $g(\boldsymbol{r}, V)$ gives rise to the temperature induced electronic structure changes. The electronic structures are measured in a wide energy range from -800 mV to 800 mV which includes the PG energy range. The cross-correlation coefficient between $g(\boldsymbol{r}, V)$ at 5 K and 55 K are around 0.9 in the large energy range (Supplementary Figure 6d). This method provides meaningful subtraction of high ($T > T_c$) and low ($T < T_c$) temperature data to detect temperature induced differences of the electronic structures at atomic scale.

Supplementary Note 4

Predicted temperature-evolution of gap map $\Delta_1(\boldsymbol{r})$

We calculated the temperature evolution of the gap map $\Delta_1(\boldsymbol{r})$. $\Delta_1(\boldsymbol{r})$ is defined as the energy of the coherence peak at $E > 0$, i.e., the same definition as the experimental measurement in main-text Figure 3. The gap modulation in the PDW+DSC state has a periodicity of $8a_0$ (Supplementary Figure 7a and b). The amplitude of the y-averaged gap modulation is $\sim 0.14t$ at $T = 0$ and $\sim 0.13t$ at $T = 0.04t$. The gap modulation in the pure PDW state has a periodicity of $4a_0$ (Supplementary Figure 7c). The amplitude of the y-averaged gap modulation is $\sim 0.05t$ at $T = 0.09t$, which is much smaller compared to the PDW+DSC state. This is a consequence of the reduction in the PDW gap order parameter with increasing temperature (Fig. 2d). In this prediction the modulation periodicity of $\Delta_1(\boldsymbol{r})$ changes from $8a_0$ to $4a_0$ in the transition from the PDW+DSC state to the pure PDW state. In experiments we have observed that $\Delta_1(\boldsymbol{r})$ modulates at $8a_0$ (inset in main-text Figure 3a) at $T \ll T_c$. However, the modulation periodicity of $\Delta_1(\boldsymbol{r})$ could not be determined at $T = 55\text{K} = 1.5T_c$ due to the presence of large regions with indeterminate coherence peaks (see white regions in Figure 3f). Therefore, the predicted temperature-evolution of the gap map $\Delta_1(\boldsymbol{r})$ could not be tested.

We note that the gap modulation is also possible in a state with coexisting charge density wave (CDW) and uniform DSC. In particular, a d-form factor (dFF) bond density wave (BDW) with wavevector $\boldsymbol{Q}_c = (\pm 1/4, 0)\frac{2\pi}{a_0}$ is often considered as a main candidate of charge order in underdoped cuprates both below and above T_c[21,22,23]. These states (Supplementary Figure 8), however, cannot account for the presence of gap modulations with wavevectors $\sim (\pm 1/8, 0)\frac{2\pi}{a_0}$ and $(0, \pm 1/8)\frac{2\pi}{a_0}$ in the experimental data (main-text Fig. 3a).

Supplementary Note 5

Bogoliubov quasiparticle scattering interference calculations

Bogoliubov quasiparticle scattering interference (BQPI) is a consequence of impurity scattering. To study the BQPI characteristics of the PDW+DSC and PDW states, we consider a point-like potential scatterer with impurity potential V_{imp} located at the lattice site i^* in the middle of an $N \times N$ square lattice. The resulting system is described by the following Hamiltonian

$$H = H_{MF} + H_{imp}, \tag{22}$$

where, H_{MF} is given by Supplementary Eq. (7), and the impurity Hamiltonian can be expressed as

$$H_{imp} = V_{imp} \sum_{\sigma} n_{i^*\sigma}, \tag{23}$$

We set $N = 56$ and $V_{imp} = 3t$. The Hamiltonian H can be diagonalized following the same procedure used for diagonalizing H_{MF}. The resulting BdG equations have the same form as the clean system [Supplementary Eq. (13)] with only difference that the onsite potentials [Supplementary Eq. (10)] are changed to $\mu_{i\sigma} \to \mu_{i\sigma} - V_{imp}\delta_{ii^*}$, where δ_{ij} is the Kronecker delta function. We solve the BdG equations self-consistently to obtain the PDW+DSC and pure PDW states in presence of an impurity. Subsequently, we compute continuum LDOS $N(\boldsymbol{r}, E)$ and thereby $Z(\boldsymbol{r}, E) = N(\boldsymbol{r}, +E)/N(\boldsymbol{r}, -E)$ for $E > 0$ using the procedure outlined in Supplementary Note 1. QPI $Z(\boldsymbol{q}, E)$ maps are obtained by taking Fourier transform of the $Z(\boldsymbol{r}, E)$ maps. Finally, energy integrated BQPI maps are obtained by summing $Z(\boldsymbol{q}, E)$ maps over the range $0 < E < \Delta_0$.

$$\Lambda_P(\boldsymbol{q}, \Delta_0) = \sum_{E\cong 0}^{\Delta_0} Z(\boldsymbol{q}, \mathrm{E}) \tag{24}$$

The upper cut-off of the energy sum is set to $\Delta_0 = 0.05t = 20$ meV ($t = 400$ meV) to match with the experiment.

The as obtained $Z(\boldsymbol{q}, E)$ maps exhibit largest intensity at PDW driven charge order Bragg peaks $\boldsymbol{q} = \pm n\boldsymbol{Q}_P, n = 0, 1, 2, \ldots, 7$ in PDW+DSC state and $\boldsymbol{q} = \pm n(2\boldsymbol{Q}_P), n = 0, 1, 2, 3$, in the pure PDW state (the fundamental charge order harmonic occurs at $\boldsymbol{Q}_c = \boldsymbol{Q}_P$ in PDW+DSC state and at $\boldsymbol{Q}_c = 2\boldsymbol{Q}_P$ in pure PDW state as explained in the main-text), see Supplementary Fig. 9a, d. Accounting for the discommensurate short-range nature of the charge order in $Bi_2Sr_2CaCu_2O_{8+\delta}$ [24] will smear the Bragg peaks and reduce their intensity. The exact amount of suppression is not clear, though. In order to emphasize the QPI wavevectors emerging from impurity scattering, we have chosen to suppress the Bragg peaks by a factor $F = 100$ (Supplementary Fig. 9b, e). Finally, the resulting $Z(\boldsymbol{q}, E)$ maps are symmetrized by adding their 90°-rotated versions to account for the orthogonal domains of unidirectional charge modulations seen in the experiments[25] (Supplementary Fig. 9c, f). To further illustrate the effects of suppression of charge order Bragg peaks we show $\Lambda_P(\boldsymbol{q}, \Delta_0)$-maps with suppression factors $F = 1, 10, 50, 100, 1000$, in Supplementary Fig. 10. Clearly, if shown to scale ($F = 1$), the Bragg peaks will obscure all wavevectors emerging from impurity scattering. We found that a better match with the experimental result can be obtained by using $F = 100$, although the qualitative features do not change significantly with F once the Bragg peaks are suppressed somewhat, around $F = 50$.

Energy integrated BQPI map $\Lambda_C(\boldsymbol{q}, \Delta_0)$ in CDW state is constructed non-self-consistently via two independent methods. The first method is setting gap order parameter in self-consistent PDW state (at $T = 0.09t$) to zero while keeping bond order and on-site potential modulations intact (Supplementary Fig. 11a and main-text Fig. 5b). The other method is taking the normal state Hamiltonian from the uniform DSC state solution (at $T = 0.09t$) and adding a term producing a d-form factor bond ordered charge density

wave (dFF-BDW) with wavevector $\boldsymbol{Q}_C = (1/4, 0)\frac{2\pi}{a_0}$ (Supplementary Fig. 11b). The amplitude of the charge density wave is set to be the same as the uniform DSC state gap field. This state becomes equivalent to that in Supplementary Fig. 8 if the coexisting DSC state in the later is removed. To calculate $\Lambda_C(\boldsymbol{q}, \Delta_0)$, an impurity Hamiltonian is added and subsequently the corresponding total Hamiltonians are diagonalized in the real space. This procedure is equivalent to a T-matrix calculation. $\Lambda_C(\boldsymbol{q}, \Delta_0)$ in the pure CDW state obtained from both methods exhibit features very different from the $\Lambda(\boldsymbol{q}, \Delta_0)$ observed in experiments. We have presented $\Lambda_C(\boldsymbol{q}, \Delta_0)$ map from Supplementary Figure 11a in the main-text Figure 5b.

Supplementary Note 6

Comparison between theoretical and experimental $\Lambda(\boldsymbol{q}, \Delta_0)$ data

Here we compare the theoretical and experimental $\Lambda(\boldsymbol{q}, \Delta_0)$ data in detail. The feature extending in the nodal directions (red arrow in Supplementary Figure 12a and c) is a signature of the uniform DSC component in the PDW+DSC state (below T_c), which disappear in the pure PDW state (above T_c). Supplementary Figure 12e-f shows superimposing the predicted $\Lambda_P(\boldsymbol{q}, \Delta_0)$ of PDW+DSC state onto the measured $\Lambda(\boldsymbol{q}, \Delta_0)$ of the superconducting phase, and superimposing the $\Lambda_P(\boldsymbol{q}, \Delta_0)$ of pure PDW state onto the $\Lambda(\boldsymbol{q}, \Delta_0)$ of the pseudogap phase, respectively. The positions of the experimental and theoretical QPI features are nearly identical.

Moreover, we measure the arc-like feature in the experimental $\Lambda(\boldsymbol{q}, \Delta_0)$ and theoretical $\Lambda_P(\boldsymbol{q}, \Delta_0)$. The extension of the arc is quantified by the angle subtended by the arc. We fit each arc of a circle about $(\pm1, \pm1)$ $2\pi/a_0$ point using least square fit (see Supplementary Figure 13). This procedure is carried out for six temperatures in both the theory and the experiment. The measured arc extension increases as a function of temperature from superconducting to pseudogap phase (Supplementary Figure 14a). This measurement agrees with the predicted arc extension in $\Lambda_P(\boldsymbol{q}, \Delta_0)$ from PDW+DSC state to pure PDW state (Supplementary Figure 14b).

Supplementary Note 7

Energy-evolution of QPI signatures of the pseudogap phase and the PDW state

To avoid the 'setup' effect in the experiments, we calculate the ratio of the total density of states

$$Z(\boldsymbol{r}, \mathrm{V}) \equiv \frac{g(\boldsymbol{r}, +\mathrm{V})}{g(\boldsymbol{r}, -\mathrm{V})} \qquad (25)$$

We take the power spectral density Fourier transform $Z(\boldsymbol{q}, \mathrm{V})$ of $Z(\boldsymbol{r}, \mathrm{V})$. The $Z(\boldsymbol{q}, \mathrm{V})$ are summed up to Δ_0, the energy that the Bogoliubov quasiparticles cease to exist[26]. Δ_0 is around 20 meV in the 8% hole-doped $Bi_2Sr_2CaDyCu_2O_8$ sample studied in this paper. The energy evolution of the experimental $Z(\boldsymbol{q}, \mathrm{V})$ maps from 8 meV to 20 meV and the corresponding calculated $Z(\boldsymbol{q}, \mathrm{V})$ maps are presented in Supplementary Figure 15. The energy evolution of the wavevectors are visualized in a supplementary movie of $Z(\boldsymbol{q}, V, 55\ \mathrm{K})$ from 2 mV to 20 mV. The wavevectors evolve dispersively with energy only by a small amount.

Legends of Additional Supplementary Files

Supplementary Movie 1. Determination of Δ_0 from a movie of $Z(\boldsymbol{q}, V)$ at $T = 4.2$ K. Δ_0 is defined as the energy that the Bogoliubov quasiparticles cease to exist.

Supplementary Movie 2. Energy evolution of quasiparticles in the pseudogap phase shown in a movie of of $Z(\boldsymbol{q}, V)$ at $T = 55$ K.

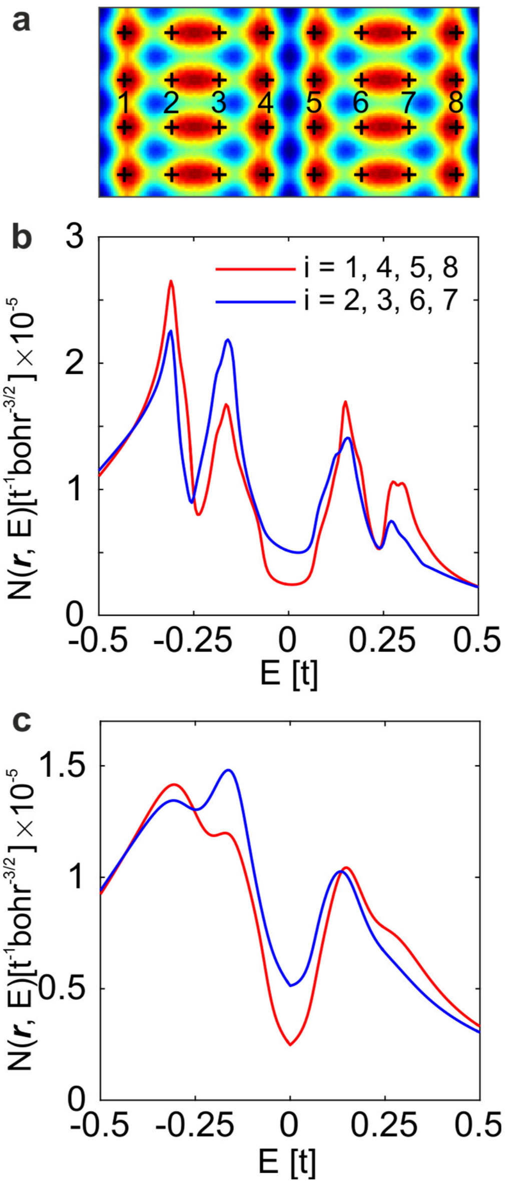

Supplementary Figure 1. Continuum LDOS in PDW state over a period of PDW ($8a_0$).

a. Continuum LDOS map $N(\boldsymbol{r}, E = \Delta_1)$ in the pure PDW state at $T = 0.09t$ over a $4a_0 \times 8a_0$ area, obtained using Supplementary Eq. (19), for the same parameter set as in Fig. 2a of the main-text. The location of a Cu atom is indicated by a black cross.

b. Continuum LDOS spectra $N(\boldsymbol{r}, E)$ above Cu positions, marked in the panel (a), without incorporating inelastic scattering.

c. Continuum LDOS spectra $N(\boldsymbol{r}, E)$ at the same positions as in (b), obtained after incorporating $\Gamma = \alpha|E|$ inelastic scattering ($\alpha = 0.25$).

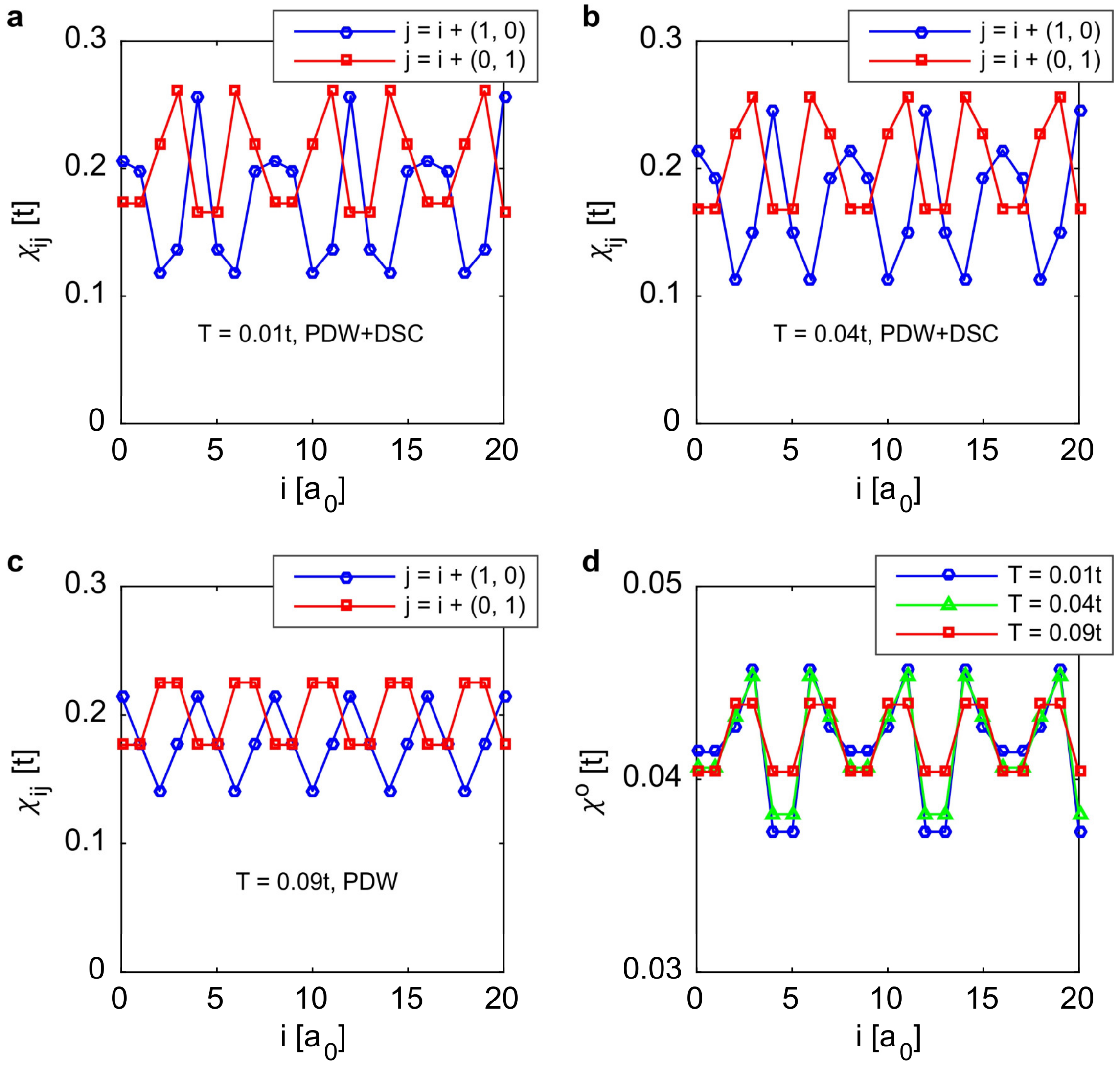

Supplementary Figure 2. Variation of bond mean-fields and bond order in PDW+DSC and pure PDW states. Nearest-neighbor bond mean-fields in PDW+DSC state at (**a**) $T = 0.01t$, (**b**) $T = 0.04t$, and (**c**) in pure PDW state at $T = 0.09t$; and (**d**) bond order parameter χ_i^o at these temperatures.

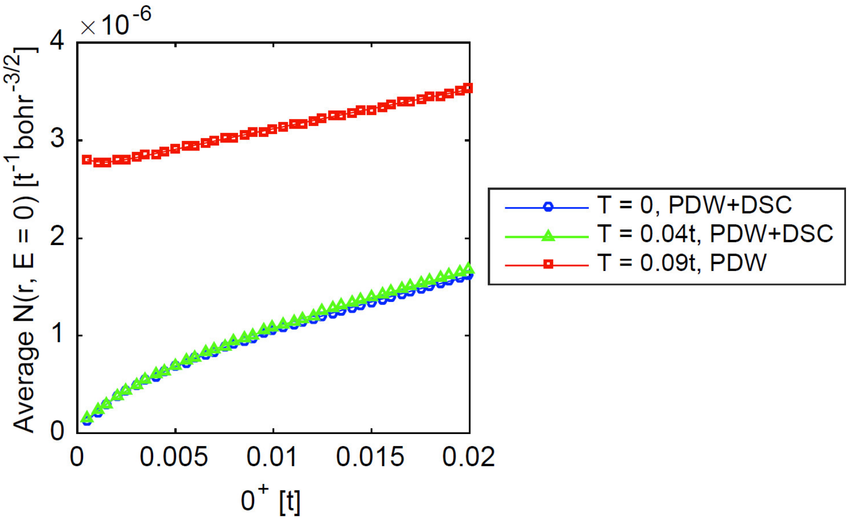

Supplementary Figure 3. Variation of average continuum LDOS at zero-energy with artificial broadening factor 0^+ employed in the calculation of the lattice Green's function [Supplementary Eq. (16)].

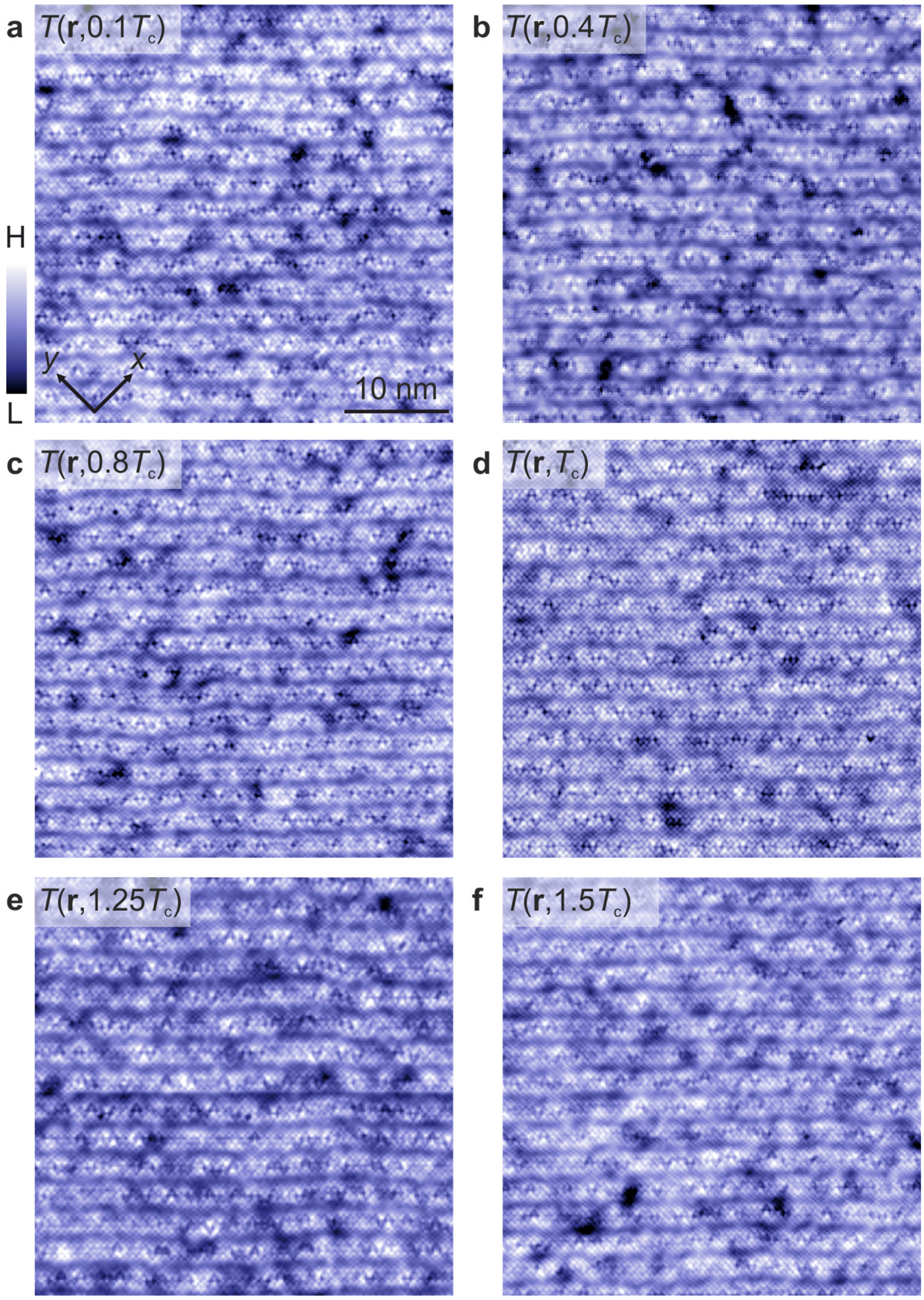

Supplementary Figure 4. Topography $T(\boldsymbol{r})$ in a 40 nm × 40 nm FOV of the underdoped $Bi_2Sr_2CaDyCu_2O_8$ sample. The six QPI $\Lambda(\boldsymbol{q},\Delta_0)$ maps from $0.1T_c$ to $1.5T_c$ are measured therein.

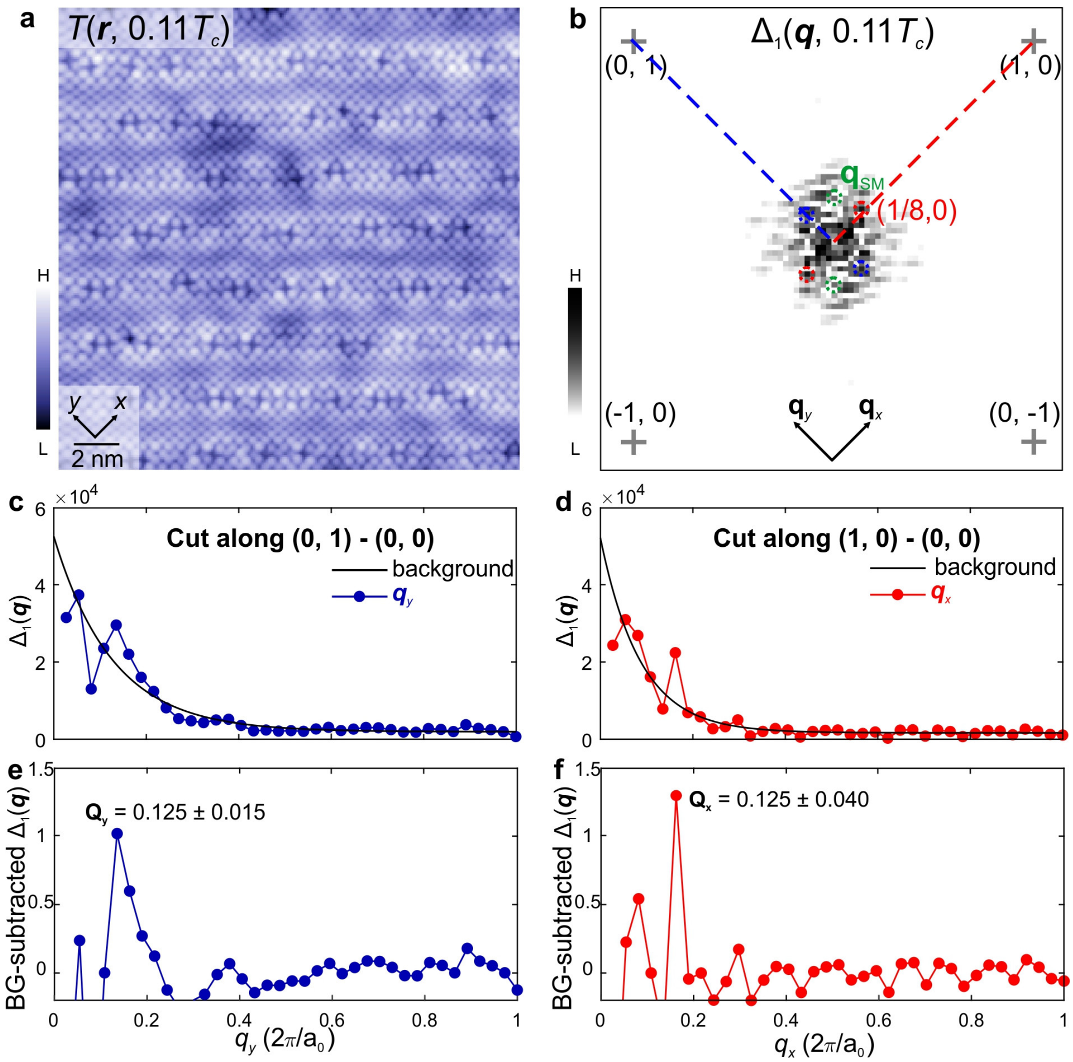

Supplementary Figure 5. Spatial variations and modulations in cuprate pseudogap energy gaps.

a. 20 nm × 20 nm topographic image $T(\boldsymbol{r})$ of BiO termination of $p \approx 8\%$ hole-doped $Bi_2Sr_2CaDyCu_2O_8$ surface at $T = 0.11T_c = 4.2$K. The gap map $\Delta_1(\boldsymbol{r})$ in Figure 3a in the main-text is taken simultaneously from this FOV.

b. Amplitude Fourier transform $\Delta_1(\boldsymbol{q})$ derived from the gap map $\Delta_1(\boldsymbol{r})$ at $T = 0.11T_c = 4.2$K (Figure 3a in the main-text). The 1/8 peaks are marked by blue and red circles. The ~1/5 peaks related to the supermodulation of the BiO termination are marked by green circles.

c & **d**. Measured $\Delta_1(\boldsymbol{q})$ along (**c**) (0,0)-(0,1) and (**d**) (0,0)-(1,0). The linecut measurements are transverse average of 2 or 3 pixels. The measurements are subsequently fitted to an exponential background.

e & **f**. The same data as **c** & **d** but with the exponential background subtracted. The intensity due to the PG gap modulation is strongest at the $\boldsymbol{Q}_p \approx (0, \pm 1/8)2\pi/a_0$ that represents eight-unit-cell gap modulations in the y direction, and $\boldsymbol{Q}_p \approx (\pm 1/8, 0)2\pi/a_0$ that represents the gap modulations in the x-direction.

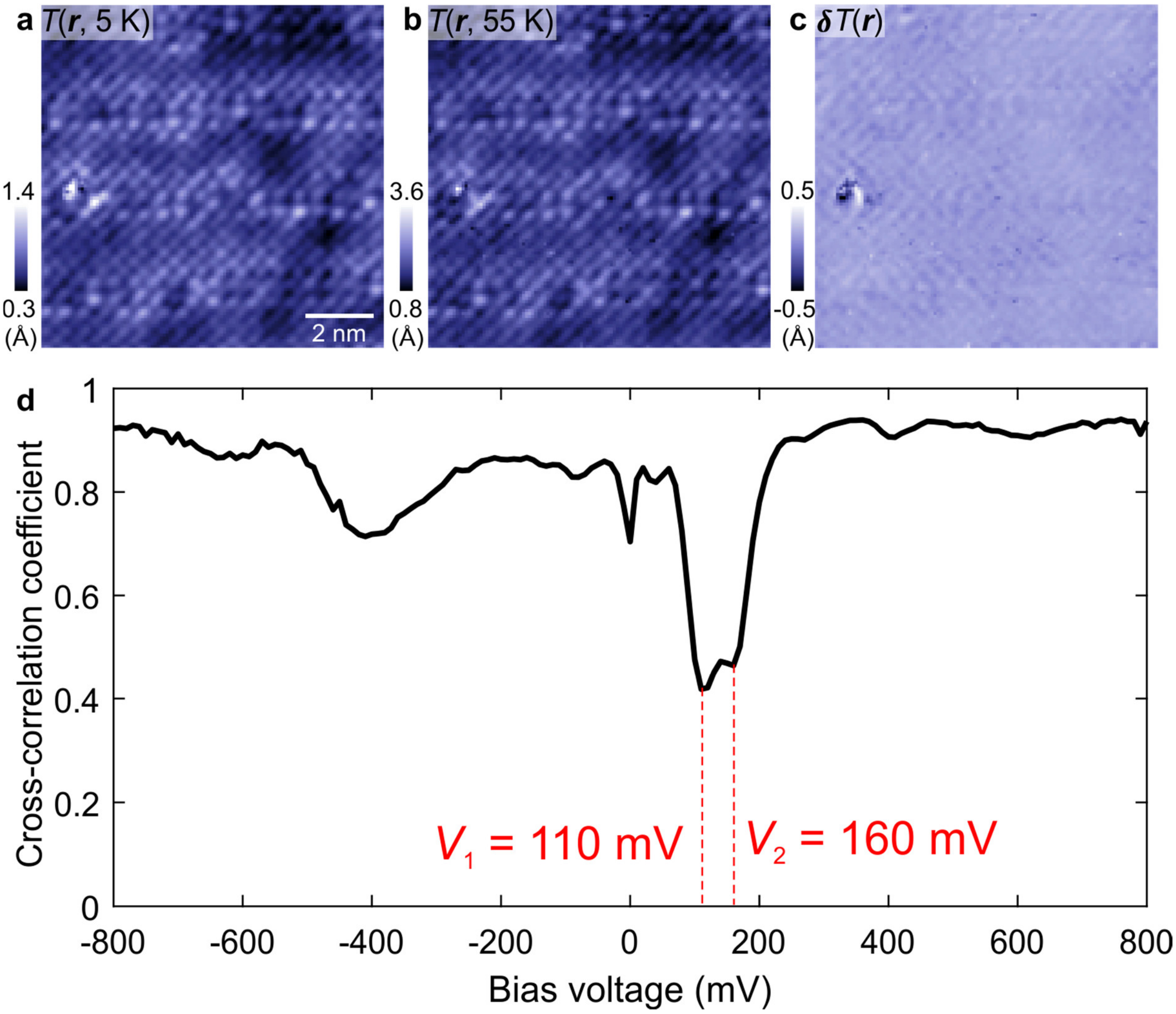

Supplementary Figure 6. Spatial registration of the two datasets at 5 K and 55 K.

a & **b**. $T(\boldsymbol{r}, 5\text{ K})$ and $T(\boldsymbol{r}, 55\text{ K})$ processed using the Lawler-Fujita algorithm. The distortions are now corrected and the CuO_2 lattice are identically periodic.

c. Measured $\delta T(\boldsymbol{r}) = T(\boldsymbol{r}, 55\text{ K}) - T(\boldsymbol{r}, 5\text{ K})$ showing the FOVs are identical.

d. Cross-correlation coefficient between $g(\boldsymbol{r}, V, 5\text{ K})$ and $g(\boldsymbol{r}, V, 55\text{ K})$ as a function of bias voltage. There is strong correspondence between the two $g(\boldsymbol{r}, V)$ maps in the large energy scale except the PG energy gap scale from 110 to 160 mV.

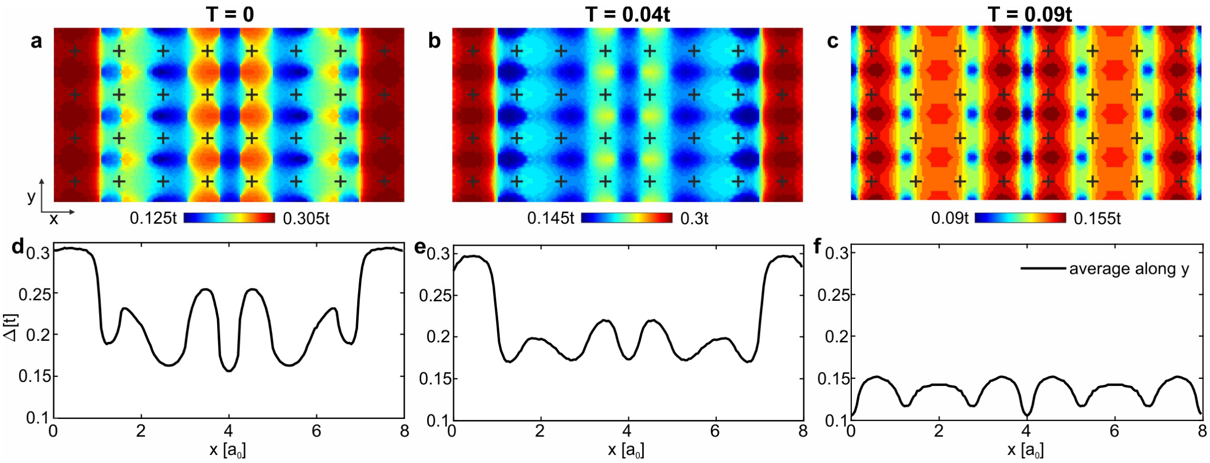

Supplementary Figure 7. Predicted temperature evolution of $\Delta_1(\boldsymbol{r})$ gap maps.

a-c. Gap maps in PDW+DSC state at (**a**) $T = 0$, (**b**) $T = 0.04t$, and in pure PDW state at (**c**) $T = 0.09t$ over $8a_0$×$4a_0$ area.

d-f. Gap averaged along y-axis (black).

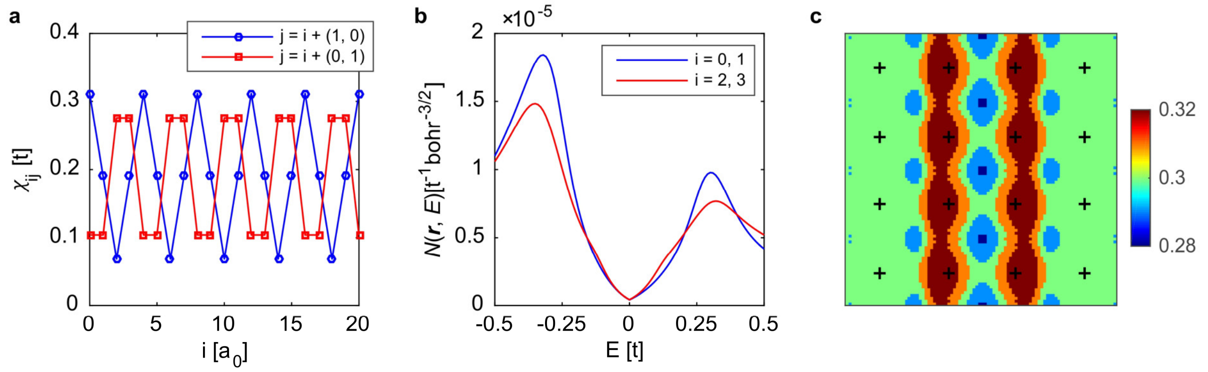

Supplementary Figure 8. Characteristics of a *d*-form factor bond density wave coexisting with uniform d-wave superconductivity (BDW+DSC).
(**a**) NN bond fields χ_{ij}, (**b**) continuum LDOS $N(\boldsymbol{r},E)$ at two inequivalent Cu sites, and (**c**) gap map $\Delta_1(\boldsymbol{r})$ (in units of t) over $4a_0$×$4a_0$ area in BDW+DSC state. The BDW+DSC state is constructed "by hand" using a model Hamiltonian which consists of the normal state term and uniform DSC term derived from the uniform DSC state solution of the RMFT t-J model at $T = 0.01t$, and d-form factor BDW term with the same amplitude as the DSC pair field, see the Supplementary Information Section D of Ref. [11].

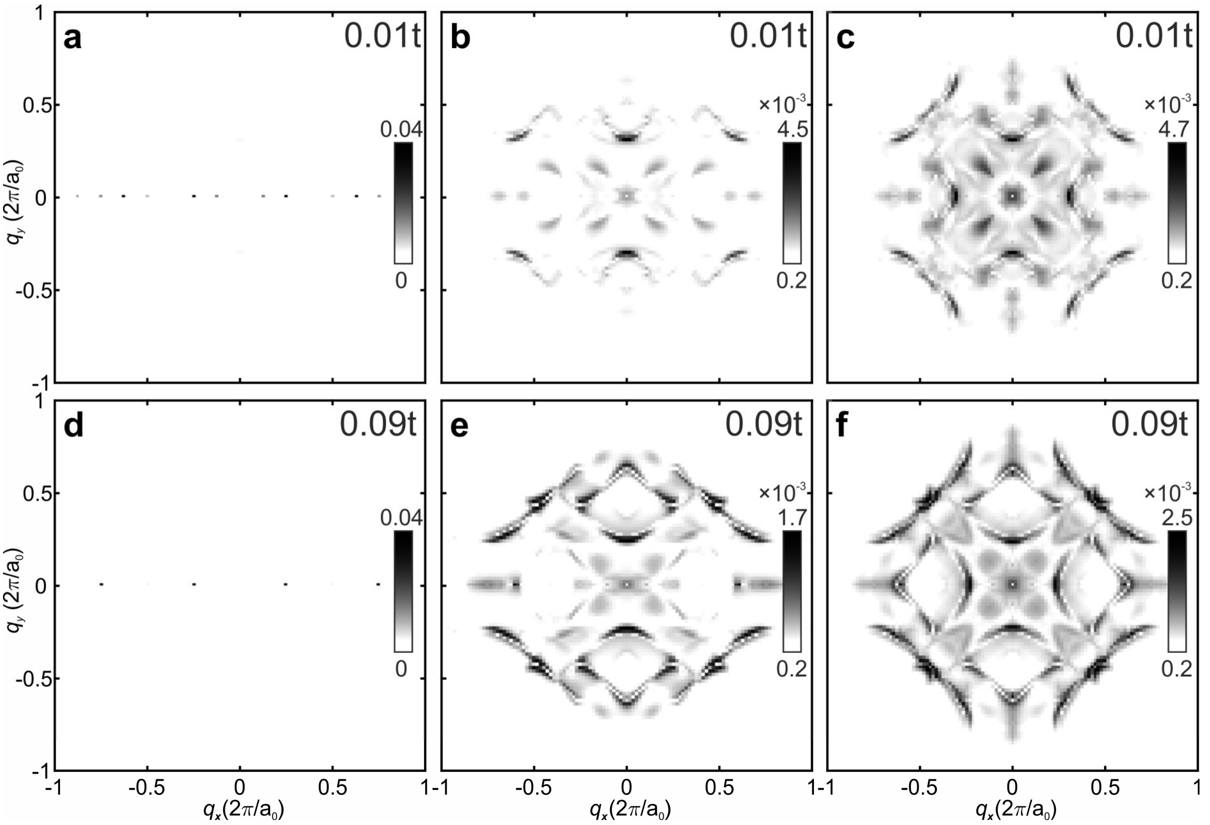

Supplementary Figure 9.
a. BQPI $Z(\boldsymbol{q}, E)$-map at energy $E = 0.03t$ in PDW+DSC state at temperature $T = 0.01t$ obtained using parameters same as in Fig. 4 of the main-text. Largest intensity occurs at non-dispersing charge order Bragg peaks $\boldsymbol{q} = \pm n\boldsymbol{Q}_P, n = 0, 1, 2, \ldots, 7$.
b. Same as in (**a**) with charge order Bragg-peaks suppressed for a better visualization of QPI wavevectors emerging from impurity scattering and to account for short-range discommensurate nature of charge order seen in the experiments.
c. Symmetrized map obtained by adding the map in (**b**) and its 90° rotated version.
d. BQPI $Z(\boldsymbol{q}, E)$-map at energy $E = 0.03t$ in pure PDW state at temperature $T = 0.09t$. Largest intensity occurs at non-dispersing charge order Bragg peaks $\boldsymbol{q} = \pm n(2\boldsymbol{Q}_P), n = 0, 1, 2, 3$.
e. Same as in (**d**) with charge order Bragg-peaks suppressed.
f. Symmetrized map obtained by adding the map in (**e**) and its 90° rotated version.

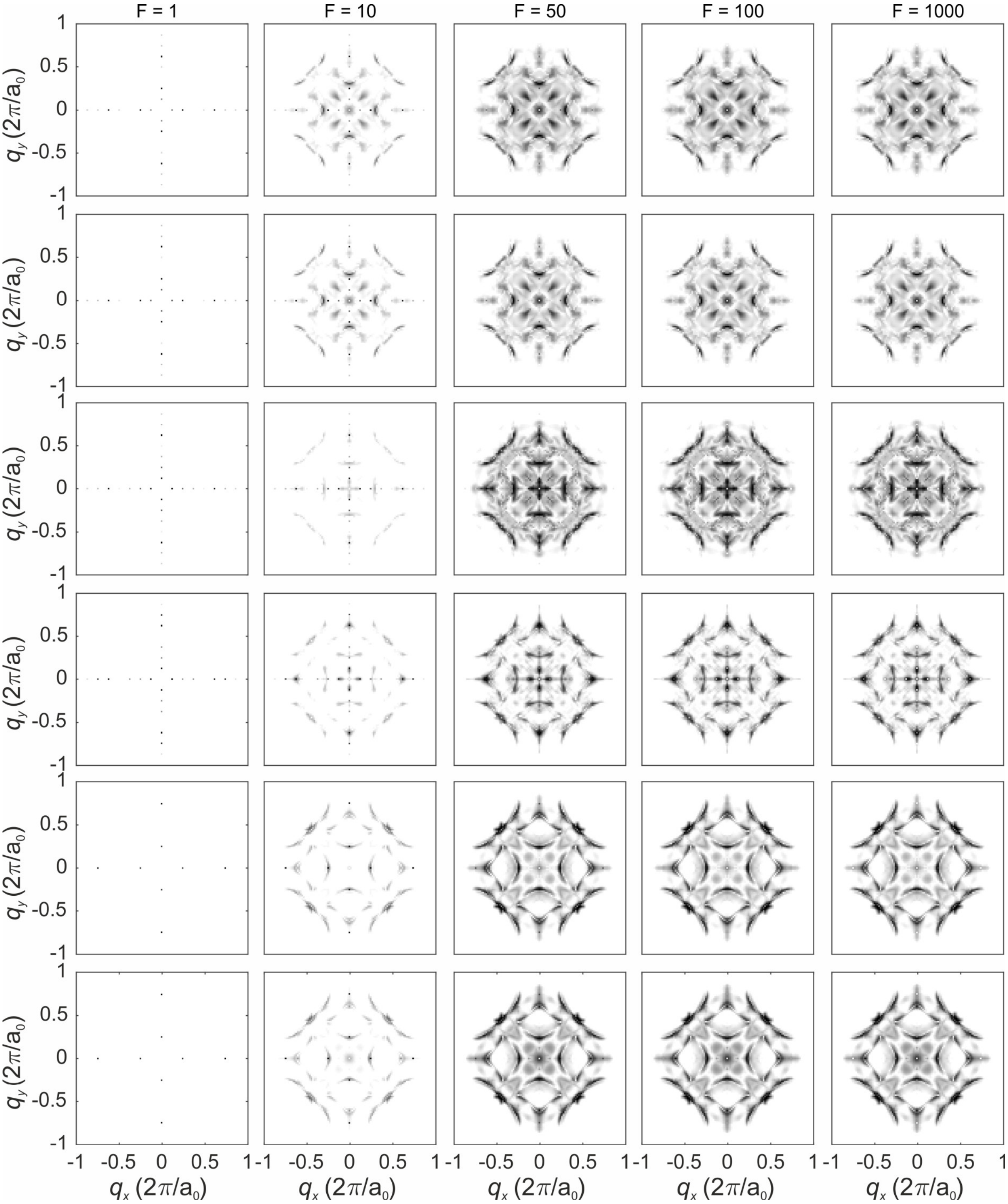

Supplementary Figure 10. Energy-integrated BQPI $\Lambda_P(\boldsymbol{q}, \Delta_0)$ in PDW+DSC state at $T = 0.01t, 0.02t, 0.04t, 0.05t$, and in pure PDW state at $T = 0.085t, 0.09t$, for various values of charge order Bragg peak suppression factors F, mentioned on the top of each column.

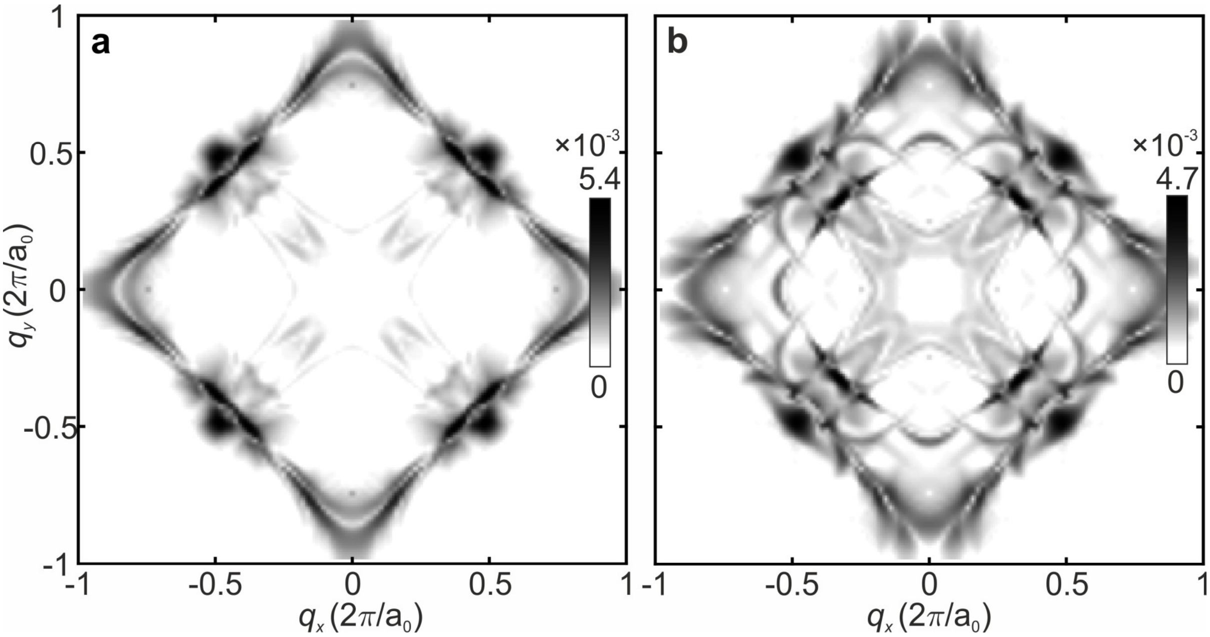

Supplementary Figure 11. $\Lambda_C(\boldsymbol{q}, 20\ \mathrm{meV})$ **for $4\boldsymbol{a_0}$ CDW state constructed non-self-consistently at temperature** $T = 0.09t$**.**

a. $\Lambda_C(\boldsymbol{q}, 20\ \mathrm{meV})$ for a CDW state constructed by setting the pair field to zero in the pure PDW state that is obtained self-consistently at $T = 0.09t$.

b. $\Lambda_C(\boldsymbol{q}, 20\ \mathrm{meV})$ for a CDW state constructed by taking the normal state Hamiltonian from the uniform DSC state solution at $T = 0.09t$ and, subsequently, adding a d-form factor charge density wave term.

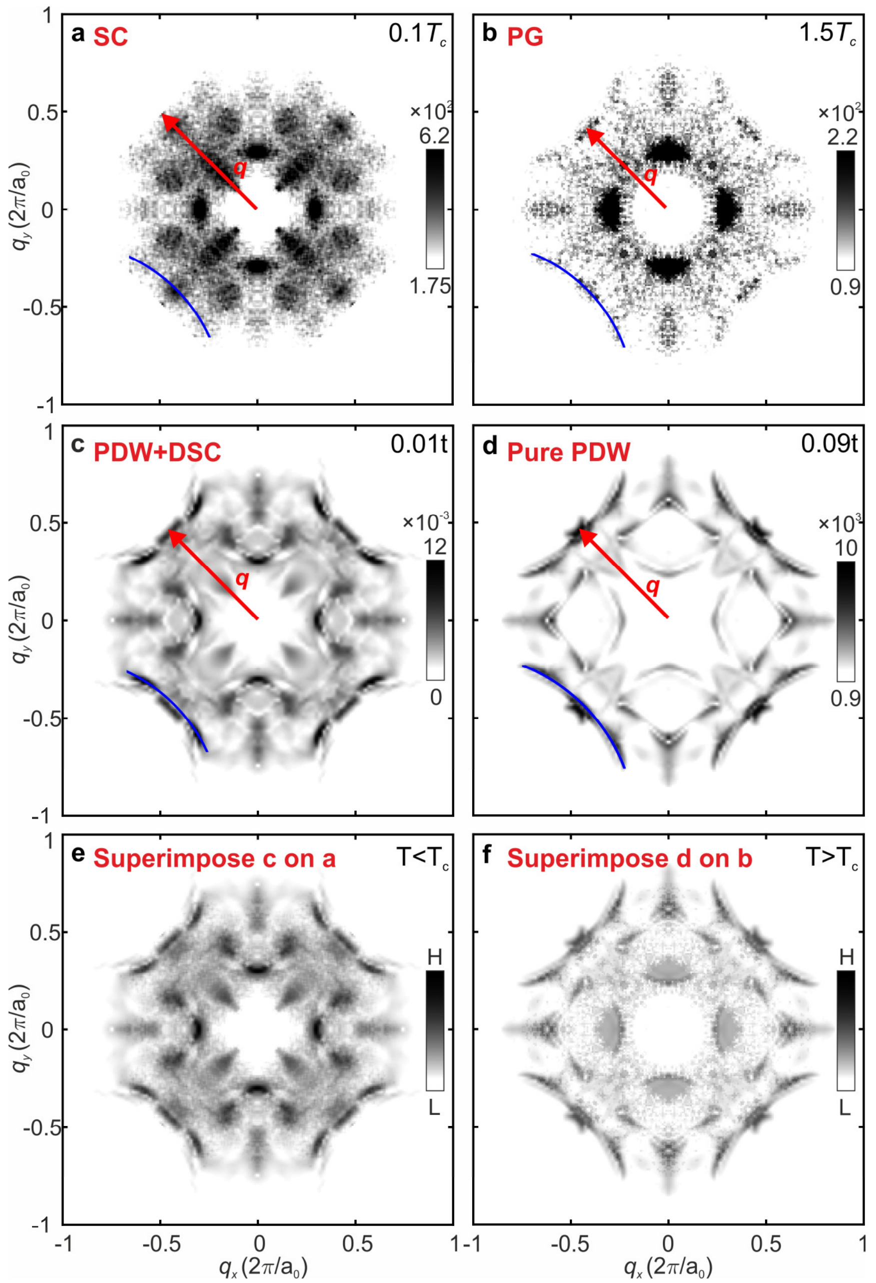

Supplementary Figure 12. Discrimination of QPI signature of the pseudogap phase from the superconducting state.

a. Measured $\Lambda(\boldsymbol{q}, 20\text{ meV})$ in the superconducting state at $T = 0.1T_c$. The contrast of the QPI pattern is maximized for better comparison between the superconducting state and the pseudogap phase. The arc is highlighted by a blue curve. The lobe extending in the nodal direction is indicated by a red arrow.

b. Measured $\Lambda(\boldsymbol{q}, 20\text{ meV})$ in the pseudogap phase at $T = 1.5T_c$.

c. Predicted $\Lambda_P(\boldsymbol{q}, 20\text{ meV})$ of the PDW +DSC state at $T = 0.01t$. The lobe extending in the nodal direction is a signature of the presence of DSC component.

d. Predicted $\Lambda_P(\boldsymbol{q}, 20\text{ meV})$ of the pure PDW state at $T = 0.09t$. The lobe in the nodal direction disappears above T_c, as a consequence of vanishing DSC component.

e-f. Superimposition of the calculated $\Lambda_P(\boldsymbol{q}, 20\text{ meV})$ onto the measured $\Lambda(\boldsymbol{q}, 20\text{ meV})$ shows excellent coincidence of the positions of dominant QPI features.

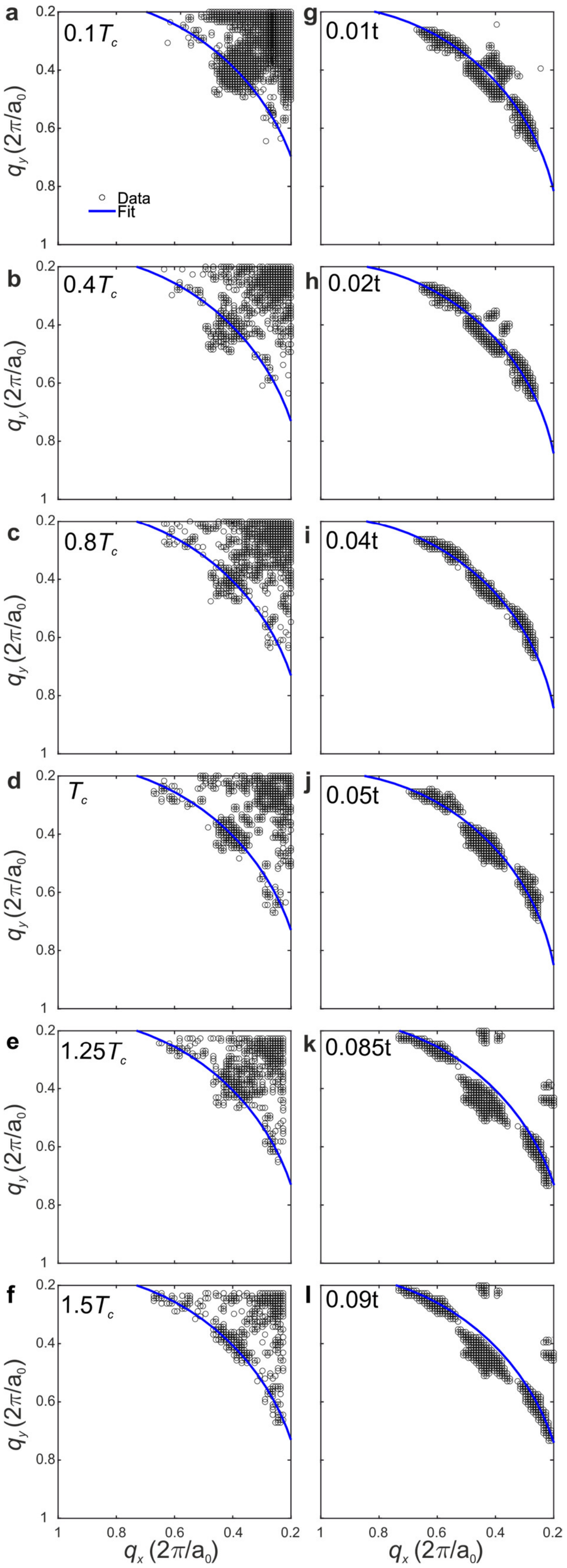

Supplementary Figure 13. The arc feature in $\Lambda(\boldsymbol{q}, 20\,\text{meV})$ is fit by a circle about $(\pm1, \pm1)\ 2\pi/a_0$ point. The angle subtended by this arc is measured versus temperature.

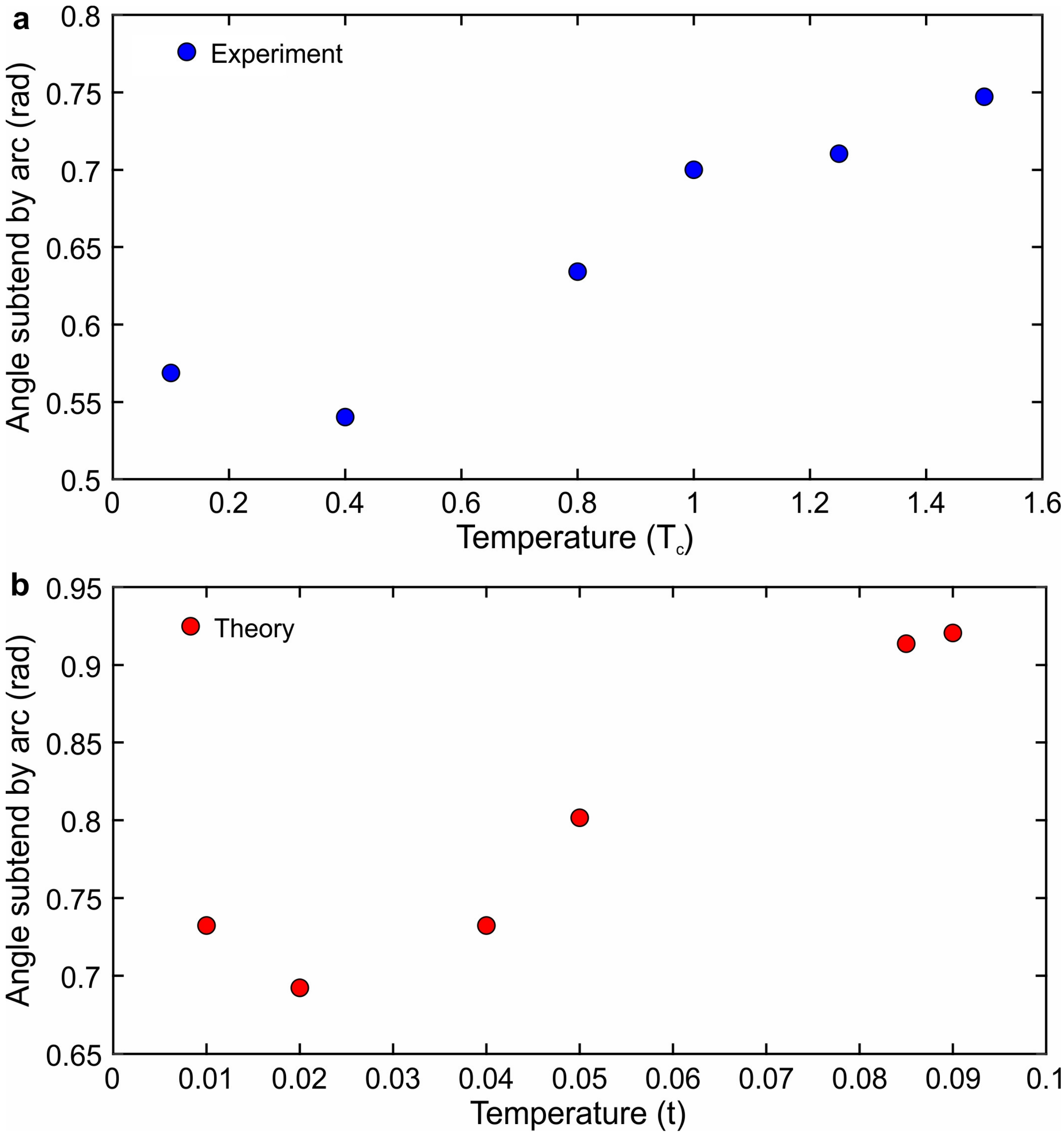

Supplementary Figure 14. The temperature dependence of the arc feature in (**a**) measured $\Lambda(\boldsymbol{q}, 20\text{ meV})$ and (**b**) predicted $\Lambda_P(\boldsymbol{q}, 20\text{ meV})$. The arc extension grows with temperature through T_c in both measurement and predictions.

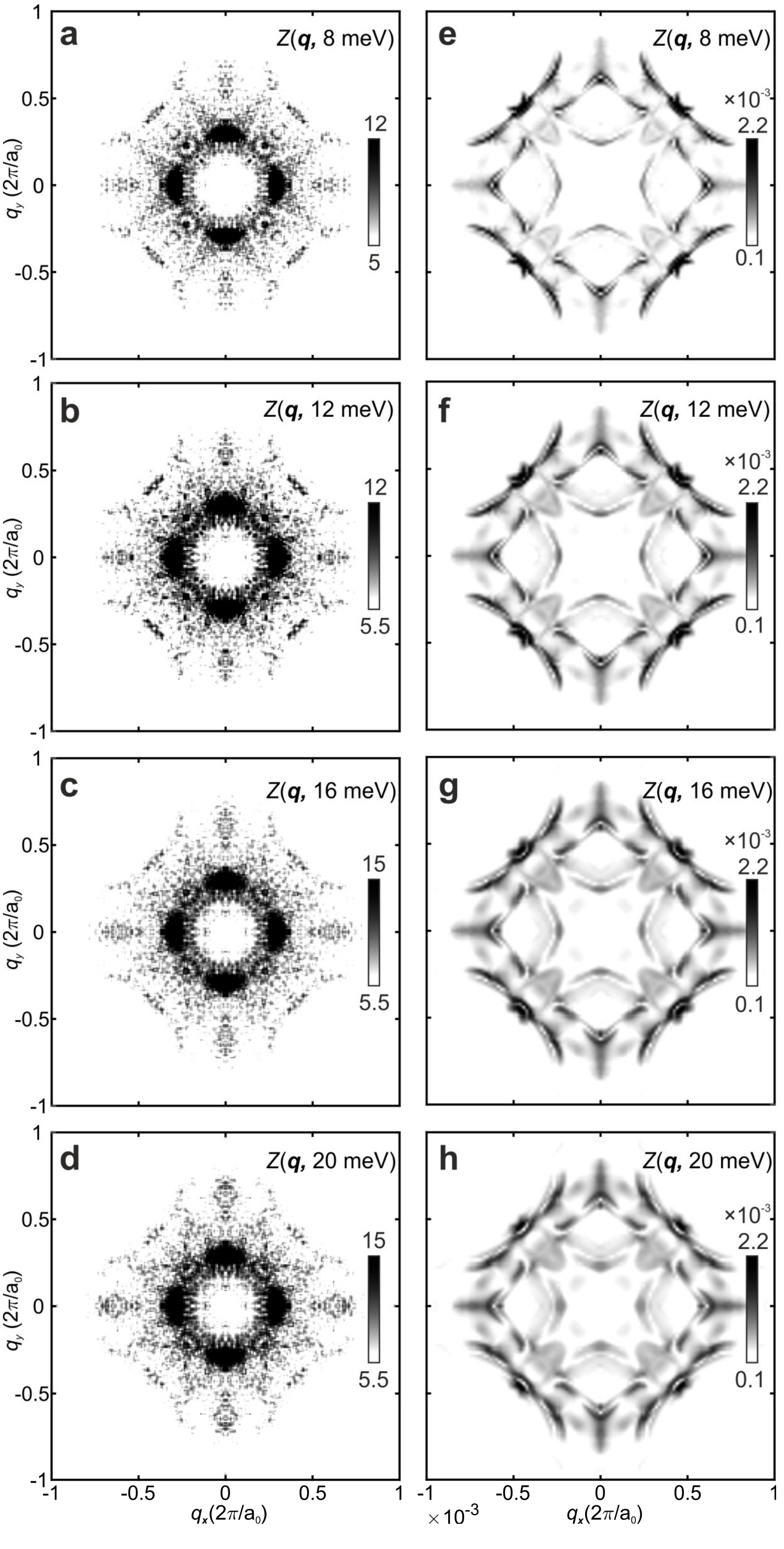

Supplementary Figure 15. Energy dependence of quasiparticle inference $Z(\boldsymbol{q}, \mathrm{V})$ in the pseudogap phase and the pure PDW state.
a-**d**. The experimental $Z(\boldsymbol{q}, \mathrm{V})$ maps measured at 55 K.
e-**h**. The theoretical $Z(\boldsymbol{q}, \mathrm{V})$ maps predicted for the pure PDW state $T = 0.09t$.

References

1 Yang, K. Y., Chen, W. Q., Rice, T. M., Sigrist, M. & Zhang, F. C. Nature of stripes in the generalized t-J model applied to the cuprate superconductors. *New J. Phys.* **11**, 055053 (2009).

2 Tu, W. L. & Lee, T. K. Genesis of charge orders in high temperature superconductors. *Sci. Rep.* **6**, 18675 (2016).

3 Choubey, P., Tu, W. L., Lee, T. K. & Hirschfeld, P. J. Incommensurate charge ordered states in the t-t′-J model. *New J. Phys.* **19**, 013028 (2017).

4 Himeda, A., Kato, T. & Ogata, M. Stripe States with Spatially Oscillating d-Wave Superconductivity in the Two-Dimensional t-t'J Model. *Phys. Rev. Lett.* **88**, 117001 (2002).

5 Raczkowski, M., Capello, M., Poilblanc, D., Frésard, R. & Oleś, A. M. Unidirectional d -wave superconducting domains in the two-dimensional t-J model. *Phys. Rev. B* **76**, 140505 (2007).

6 Corboz, P., Rice, T. M. & Troyer, M. Competing states in the t - J model: Uniform d-wave state versus stripe state. *Phys. Rev. Lett.* **113**, 046402 (2014).

7 Hamidian, M. H. *et al.* Detection of a Cooper-pair density wave in $Bi_2Sr_2CaCu_2O_{8+x}$. *Nature* **532**, 343–347 (2016).

8 Edkins, S. D. *et al.* Magnetic field-induced pair density wave state in the cuprate vortex halo. *Science* **364**, 976–980 (2019).

9 Du, Z. *et al.* Imaging the energy gap modulations of the cuprate pair-density-wave state. *Nature* **580**, 65–70 (2020).

10 Christensen, R. B., Hirschfeld, P. J. & Andersen, B. M. Two routes to magnetic order by disorder in underdoped cuprates. *Phys. Rev. B* **84**, 184511 (2011).

11 Choubey, P. *et al.* Atomic-scale Electronic Structure of the Cuprate Pair Density Wave State Coexisting with Superconductivity. *Proc. Natl. Acad. Sci. U. S. A.* **117**, 14805–14811 (2020).

12 Ogata, M. & Himeda, A. Superconductivity and Antiferromagnetism in an Extended Gutzwiller Approximation for t-J Model: Effect of Double-Occupancy Exclusion. *J. Phys. Soc. Japan* **72**, 374 (2003)

13 Tersoff, J. & Hamann, D. R. Determination of the lengths of nonisotropic linear features in micrographs. *Phys. Rev. Lett.* **50**, 1998 (1983).

14 Choubey, P., Berlijn, T., Kreisel, A., Cao, C. & Hirschfeld, P. J. Visualization of atomic-scale phenomena in superconductors: Application to FeSe. *Phys. Rev. B.* **90**, 134520 (2014).

15 Choubey, P., Kreisel, A., Berlijn, T., Andersen, B. M. & Hirschfeld, P. J. Universality of scanning tunneling microscopy in cuprate superconductors. *Phys. Rev. B* **96**, 174523 (2017).

16 Kreisel, A. *et al.* Interpretation of scanning tunneling quasiparticle interference and impurity states in cuprates. *Phys. Rev. Lett.* **114**, 217002 (2015).

17 Alldredge, J. W. *et al.* Evolution of the electronic excitation spectrum with strongly diminishing hole density in superconducting $Bi_2Sr_2CaCu_2O_{8+\delta}$. *Nat. Phys.* **4**, 319–326 (2008).

18 Baruch, S. & Orgad, D. Spectral signatures of modulated d -wave superconducting phases. *Phys. Rev. B* **77**, 174502 (2008)

19 Liu, X., Chong, Y. X., Sharma, R. & Davis, J. C. S. Discovery of a cooper-pair density wave state in a transition-metal dichalcogenide. *Science* **271**, 1447-1452 (2021).

20 Lawler, M. J. *et al.* Intra-unit-cell electronic nematicity of the high-T_c copper-oxide pseudogap states. *Nature* **466**, 347–351 (2010).

21 Sachdev, S. & La Placa, R. Bond order in two-dimensional metals with antiferromagnetic exchange interactions. *Phys. Rev. Lett.* **111**, 027202 (2013).

22 Wang, Y. & Chubukov, A. Charge-density-wave order with momentum ($2\boldsymbol{Q}$,0) and (0,$2\boldsymbol{Q}$) within the spin-fermion model: Continuous and discrete symmetry breaking, preemptive composite order, and relation to pseudogap in hole-doped cuprates. *Phys. Rev. B.* **90**, 035149 (2014).

23 Efetov, K. B., Meier, H. & Pépin, C. Pseudogap state near a quantum critical point. *Nat. Phys.* **9**, 442–446 (2013).

24 Mesaros, A. *et al.* Commensurate $4a_0$-period charge density modulations throughout the $Bi_2Sr_2CaCu_2O_{8+x}$ pseudogap regime. *Proc. Natl. Acad. Sci. U. S. A.* **113**, 12661–12666 (2016).

25 Hamidian, M. H. *et al.* Atomic-scale electronic structure of the cuprate d-symmetry form factor density wave state SI. *Nat. Phys.* **12**, 150–156 (2016)

26 Kohsaka, Y. *et al.* How Cooper pairs vanish approaching the Mott insulator in $Bi_2Sr_2CaCu_2O_{8+\delta}$. *Nature* **454**, 1072–1078 (2008).
