# Supplementary material for: Scattering interference signature of a pair density wave state in the cuprate pseudogap phase
Source: Nat Commun. 2021 Oct 19;12:6087. doi: 10.1038/s41467-021-26028-x (PMC8526682; doi:10.1038/s41467-021-26028-x)
Supplement: Supplementary file 2 — Description of Additional Supplementary Files [file 41467_2021_26028_MOESM2_ESM.pdf]

Description of Additional Supplementary Files

Supplementary Movie 1. Determination of Δ_0 from a movie of $Z(\boldsymbol{q}, V)$ at $T = 4.2$ K. Δ_0 is defined as the energy that the Bogoliubov quasiparticles cease to exist.

Supplementary Movie 2. Energy evolution of quasiparticles in the pseudogap phase shown in a movie of of $Z(\boldsymbol{q}, V)$ at $T = 55$ K.
